# A simple retarding-potential time-of-flight mass spectrometer for electrospray propulsion diagnostics

**DOI:** 10.1007/s44205-023-00045-y

**Published:** 2023-03-31

**Authors:** Christopher T. Lyne, Miron F. Liu, Joshua L. Rovey

**Affiliations:** Department of Aerospace Engineering, University of Illinois Urbana-Champaign, Urbana, IL 61801 USA

**Keywords:** Electrospray propulsion, Time-of-flight, Plume diagnostics, EMI-Im

## Abstract

The time-of-flight mass spectrometer (ToF-MS) is a useful tool for quantifying the performance of electrospray thrusters and characterizing their plumes. ToF-MS data can be used to calculate the mass-to-charge distribution in the plume, but the kinetic-energy-to-charge (i.e., the potential) distribution must be known first. Here we use a ToF-MS in tandem with a retarding potential (RP) analyzer. By sweeping the retarding potential through the range of potentials present in the plume, both the mass-to-charge distribution and the potential distribution can be measured independently. We demonstrate this technique in a case study using a capillary electrospray emitter and the ionic liquid propellant 1-Ethyl-3-methylimidazolium bis(trifluoromethylsulfonyl)imide, abbreviated EMI-Im. We report a linear correlation between retarding potential and mass-to-charge ratio that agrees with published data from more complex orthogonal RP/ToF-MS instruments. Calculated values for the jet velocity and jet breakup potential match within 2% and 12%, respectively. Using conventional ToF-MS, we estimated the propellant flow rate and compared those estimates to direct flow rate measurements. For flow rates between 233 pL/s and 565 pL/s, the error in ToF-based flow rate estimates ranged from -16% to -13% when the plume potential was assumed to be a function of mass-to-charge. Assuming a constant plume potential yielded mixed results. However, using the average stopping potential measured by a retarding potential analyzer resulted in higher errors, ranging from -26% to -30%. Data and MATLAB code are included as supplemental materials so that readers can easily apply the techniques described here.

**Supplementary Information:**

The online version contains supplementary material available at 10.1007/s44205-023-00045-y.

## Introduction

Electrospray propulsion is a type of electric propulsion in which charged droplets and ions are extracted from a liquid meniscus and accelerated by an electrostatic field. Each electrospray emitter contributes only a small amount of thrust (~10^-6^ N) at a low flow rate ($\le \sim 1 \mu g/s$), making direct measurements of thruster performance difficult. Instead, the thrust and propellant flow rate are often inferred by measuring the distributions of mass-to-charge and kinetic energy per charge (i.e., retarding potential) in the electrospray plume. This method has been used for porous [1–4], externally wetted [5–7], and capillary [8–11] electrospray thrusters. Measuring the mass-to-charge distribution can be challenging, particularly for capillary electrosprays, which emit charged droplets and ions ranging from 10^2^ to 10^6^ amu/q. Most commercial mass spectrometers (quadrupole mass spec., for instance) are intended for heavy ion analysis, with a measurable mass-to-charge range extending up to perhaps several thousand amu/q. Consequently, the preferred method for measuring the mass-to-charge distribution in capillary electrospray plumes is time-of-flight mass spectrometry, which has no upper limit on measurable mass-to-charge.

Time-of-Flight Mass Spectrometry (ToF-MS) is a technique that infers mass-to-charge from the flight time of charged particles through a fixed distance [11–13]. That is, ToF-MS measures the *velocity* of particles in the beam, which is proportional to ${\phi }^{1/2}$ and ${\zeta }^{-1/2}$, where $\phi$ is the retarding potential and $\zeta$ is the mass-to-charge ratio. For monoenergetic beams ($\phi =const.$), obtaining the mass-to-charge distribution from ToF-MS data is simple. However, for beams where there is a large potential spread or the potential distribution is not known, measuring time-of-flight is not sufficient to obtain the mass-to-charge distribution. For this general case, the ToF-MS can operate in tandem with a retarding potential (RP) analyzer. The retarding potential analyzer acts as an energy filter [7, 9, 14], selecting which particle energies are allowed into the ToF-MS. Typically, a narrow range of potentials are allowed into the ToF-MS at a given time. A mass spectrum is computed for each potential range by approximating the potential as constant. The mass spectrum of the full beam can be found by summing the mass spectra for all potentials present in the beam. This technique, known as retarding potential time-of-flight mass spectrometry (RP/ToF-MS) or sometimes known as time-of-flight stopping potential (ToF-SP), is useful for studying beams with broad energy and mass-to-charge distributions [15–17]. The key advantage of RP/ToF-MS over conventional ToF-MS is that no assumptions need to be made about the potential distribution of species in the beam. Rather, the distributions of potential and mass-to-charge are each independently measured.

Retarding potential time-of-flight mass spectrometry has been used to study capillary electrosprays. Early examples include Lozano [12] and Gamero-Castaño [18], who each used a retarding potential analyzer with a linear time-of-flight mass spectrometer. In these early works, the authors primarily used these data to comment on the relative energies of the ion and droplet populations in the plume. At that time, they did not compute a composite mass spectrum and they did not report detailed measurements of the potential distribution as a function of mass-to-charge.

More recently, RP/ToF-MS has been used to perform detailed studies of the energy and mass-to-charge distributions in capillary electrospray plumes [16, 17]. Unlike the earlier works, the instruments used in these studies are complex and require careful alignment to achieve adequate signal levels. For example, Miller et al. describe a RP/ToF-MS in which the beam travels into a DC retarding potential section, where an intermittent pulse is used to accelerate species orthogonally [17]. Species with potentials near the DC retarding potential travel into the orthogonal ToF-MS section, where they encounter additional focusing optics, a reflectron, and a multichannel plate (MCP) ion detector. The use of pulsed orthogonal extraction introduces an energy spread into the extracted beam on the order of several hundred volts, which can be partially corrected by the reflectron. The complex flight path that the beam takes through the instrument results in a poor overall transparency, causing low signal levels at the detector. To maximize signal level, Miller et al. use an einzel lens to focus the primary beam, a second einzel lens for the orthogonally extracted beam, and a relatively expensive ion detector. The operating procedure requires each einzel lens to be adjusted individually to optimally focus and align the beam for maximum signal strength. The complexity of this method also introduces uncertainty into data analysis. For instance, the range of potentials that are successfully extracted from the primary beam is determined by the ion optics of the instrument. Through extensive modeling efforts, Miller et al. estimate that species with potentials spanning 25 V are included in each scan. Ideally, this potential range would be directly controllable. For example, decreasing the range to 10 V would improve energy resolution, and increasing the range to 50 V would increase signal levels. However, their instrument does not offer direct control over the range of potentials included in a scan. Another RP/ToF-MS is described by Gamero-Castaño et al. and is significantly less complex [16]. Their RP/ToF-MS uses an electrostatic mirror for energy filtering. When the beam encounters the mirror, species within a narrow range of potentials are reflected at 90° and directed into a ToF-MS section. Like the instrument described by Miller et al., the range of potentials that are allowed into the ToF-MS section is determined by the ion optics and cannot be easily adjusted. Furthermore, the detector currents reported for this instrument are on the order of single picoamps for a beam current of several hundred nanoamps. The task of accurately measuring fast (MHz) picoamp-level signals is not trivial, and requires careful attention to electromagnetic shielding, measurement electronics, and signal processing.

These two recent examples of RP/ToF-MS instruments in the electrospray propulsion literature share two significant limitations. First, they are complicated to build and operate, requiring careful alignment and adjustments to maximize the measured signal. Second, they do not provide direct control over the range of potentials included in a measurement. Here, we present a simple RP/ToF-MS to address these deficiencies. Our RP/ToF-MS combines a linear ToF-MS with a linear retarding potential analyzer (RPA). The RPA acts as a high-pass filter, allowing species with potentials above the applied retarding potential to enter the ToF-MS. A RP/ToF-MS measurement is made by calculating the difference between ToF-MS signals at two different retarding potentials. For example, taking the difference between ToF-MS signals at retarding potentials ${\phi }_{1}$ and ${\phi }_{2}$ yields the RP/ToF-MS signal for species with potentials between ${\phi }_{1}$ and ${\phi }_{2}$ (i.e., ${\phi }_{1}<{\phi }_{RP}<{\phi }_{2}$). The range of potentials included can be directly controlled by choice of retarding potentials ${\phi }_{1}$ and ${\phi }_{2}$. Furthermore, the linear design is simple to operate and requires minimal alignment. To demonstrate the capabilities of the linear RP/ToF-MS, we present a case study of an EMI-Im capillary electrospray. First, operating in ToF-MS mode, we investigate the accuracy of time-of-flight-based propellant flow rate measurements under various assumed beam potentials. Next, we operate with retarding potential and time-of-flight in tandem (i.e., RP/ToF-MS mode) to obtain the mass-to-charge distribution in the beam without making assumptions about the beam potential distribution. Jet breakup parameters calculated from our RP/ToF-MS data match published values to within 2% for jet velocity and 12% for jet breakup potential. This case study demonstrates that the linear RP/ToF-MS can effectively resolve the energy and mass-to-charge distributions in a capillary electrospray beam, producing data comparable to more complex instruments in the literature.

## Background

### An Introduction to Basic Time-of-Flight Mass Spectrometry (ToF-MS)

This section gives a brief overview of the application of the time-of-flight technique for measuring mass-to-charge in electrospray beams. This topic is covered in more detail elsewhere, for example in [19]. ToF-MS is a technique that relies on measuring the flight time of charged particles as they drift through a region of fixed length, thereby measuring their velocities. For a particle with charge $q$ and a retarding potential ${\phi }_{RP}$, the flight time can be calculated from Eq. (1). Rearranging, we find an expression for the mass-to-charge, Eq. (2). Note that in this article, we use ‘retarding potential’ (${\phi }_{RP}$) and ‘potential’ ($\phi$) interchangeably. Each simply refers to the kinetic energy per charge of species in the electrospray plume. Thus, the plume ‘potential distribution’ describes how kinetic energy is distributed amongst the different species in the plume.

1 $$t=L\sqrt{\frac{\zeta }{2{\phi }_{RP}}}$$

2 $$\zeta =2{\phi }_{RP}\frac{{t}^{2}}{{L}^{2}}$$

The time-of-flight signal consists of the time-dependent collector current, ${I}_{C}\left(t\right)$. For a beam comprised of charged particles with potential ${\phi }_{RP}=const.$, a cumulative mass spectrum can be obtained by plotting ${I}_{C}\left(t\right)$ and transforming the x-axis from time to mass-to-charge using Eq. (2). The population distribution of mass-to-charge in the beam is obtained by differentiating the collector current with respect to time and plotting it against mass-to-charge.

The thrust and propellant mass flow rate can be calculated using Eq. (3) and Eq. (4) by scaling the time-of-flight collector current to the full beam current, ${I}^{^{\prime}}\left(t\right)={I}_{C}\left(t\right)*{I}_{B}/{I}_{C}(t=0)$ [8].

3 $$T={\int }_{0}^{\infty }2{\phi }_{RP}\left(\frac{t}{L}\right){I}^{^{\prime}}\left(t\right) dt$$

4 $$\dot{m}={\int }_{0}^{\infty }2{\phi }_{RP}{\left(\frac{t}{L}\right)}^{2}{I}^{^{\prime}}\left(t\right) dt$$

This approach can be extended to non-monoenergetic beams by expressing the beam potential as a function of flight time, ${\phi }_{RP}=f(t)$, so the integrals in Eq. (3) and Eq. (4) can be evaluated. For capillary electrosprays of EMI-Im, experimental data suggest that there is a linear relationship between retarding potential and average mass-to-charge, ${\phi }_{RP}=f(\overline{\zeta })$ [16, 17]. Plugging ${\phi }_{RP}=f(\overline{\zeta })$ into Eq. (1), the flight time can be found as a function of mass-to-charge. The potential can be converted to a function of time by associating each $\phi (\overline{\zeta })$ with its corresponding flight time. Then, Eq. (3) and Eq. (4) can be evaluated using ${\phi }_{RP}={\phi }_{RP}(t)$ to compute the thrust and propellant mass flow rate for the assumed retarding potential distribution. Similarly, the cumulative mass spectrum and population distribution can be found for the assumed retarding potential distribution by transforming the x axis from time to mass-to-charge using Eq. (2) with ${\phi }_{RP}={\phi }_{RP}(\overline{\zeta })$.

### An Introduction to Retarding Potential Time-of-Flight Mass Spectrometry (RP/ToF-MS)

An important limitation of conventional time-of-flight mass spectrometry is that the flight time depends on both the mass-to-charge and on the retarding potential, as expressed in Eq. (1). The convolution between retarding potential and mass-to-charge introduces error into measurements of the mass-to-charge distribution, thrust, and mass flow rate. This error can be minimized in conventional ToF-MS by assuming a realistic potential distribution. For example, capillary electrospray data can be analyzed using $\phi =f(\overline{\zeta })$ rather than $\phi =const$ in Eqs. (2), (3), and (4). However, this approach can’t be used if the potential distribution is not known. For this general case, ToF-MS can be used in tandem with a retarding potential (RP) analyzer, which is used to select the range of potentials allowed into the ToF-MS section. By selecting a narrow range of potentials, ToF-MS data can be analyzed assuming a constant potential without introducing significant error. A mass-to-charge spectrum for the full beam can be found by sweeping the retarding potential through the range of potentials present in the beam, measuring the spectrum for each, and then combining those into a composite spectrum. This composite spectrum accurately represents the distribution of mass-to-charge in the beam without the errors introduced by assuming a potential distribution as in conventional ToF-MS.

**Fig. 1:**
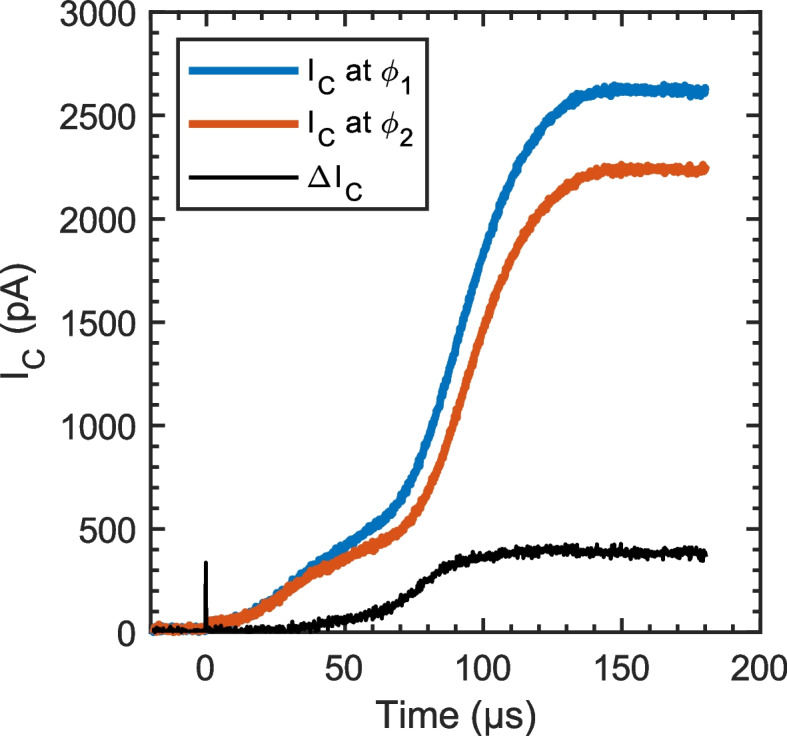
Typical time-of-flight data (colored curves), taken at different retarding potentials, after the ToF gate opens at ${\varvec{t}}=0$. The RP/ToF-MS signal (black curve) is their difference, representing the ToF signal associated with beam potentials ${{\varvec{\phi}}}_{1}\le{\varvec{\phi}}\le {{\varvec{\phi}}}_{2}$. Each time-of-flight curve represents 25,600 time-of-flight signals averaged together

Figure 1 shows typical time-of-flight signals taken at two different retarding potentials, ${\phi }_{1}$ and ${\phi }_{2}$. At $t=0$ the ToF gate is opened, and the collector current rises as charged particles in the beam begin to reach the collector. Subtracting the time-of-flight signal taken at ${\phi }_{1}$ from that at ${\phi }_{2}$, we obtain the RP/ToF-MS signal associated with beam potentials between ${\phi }_{1}$ and ${\phi }_{2}$. The mass-to-charge distribution for species with potentials ${\phi }_{1}<\phi <{\phi }_{2}$ is then calculated by transforming the x axis using Eq. (2) with ${\phi }_{RP}\approx ({\phi }_{1}+{\phi }_{2})/2$.

Note that the method used here for energy filtering the beam is somewhat atypical. Most RP/ToF-MS instruments in the literature use electrostatic energy analyzers that operate as bandpass filters for beam potential, whereas the retarding potential analyzer used here operates as a high pass filter. Thus, our method requires that two ToF-MS signals be measured, and the RP/ToF-MS signal is found by taking their difference. However, this design is simple to construct and operate, and has minimal alignment requirements. The transparency of our linear RP/ToF-MS design is also fairly high, resulting in a higher signal level as compared to more complex orthogonal RP/ToF-MS designs. For example, the maximum RP/ToF-MS signal magnitudes published by Gamero-Castaño et al. are on the order of 10 pA for a comparable electrospray beam [16]. In contrast, the maximum RP/ToF-MS signal magnitude reported here is about 400 pA. In both cases the signal levels could be increased using electrostatic beam focusing, but we expect their ratio to stay roughly constant. The linear design also allows the user to directly select the range of potentials included in each RP/ToF-MS scan. Depending on the experimental requirements, signal level can be increased by selecting a larger potential range for each scan (e.g., ${\phi }_{2}-{\phi }_{1}=50 V$) or resolution can be improved by using a smaller potential range (e.g., ${\phi }_{2}-{\phi }_{1}=5 V$).

### Calculation of mass flow rate from angle-resolved time-of-flight data

Section 2.1 described the standard method for determining the mass flow rate and thrust from time-of-flight data using Eq. (4) and Eq. (3), respectively. Those equations assume that the time-of-flight signal being analyzed is representative of the full beam, neglecting spatial variation. However, the composition of EMI-Im capillary electrospray beams varies considerably with angle [9, 19]. It is therefore worth considering how time-of-flight data taken as a function of angle can be analyzed and combined to determine the overall thrust and mass flow rate.

A simple method for obtaining the propellant mass flow rate from spatially-resolved time-of-flight data, which has been covered previously [19], approximates the mass flow rate as the beam current times the sum over all beam angles of the product of the current fraction ${f}_{C}\left(\theta \right)$, average mass-to-charge $\overline{\zeta }\left(\theta \right)$, and $\mathrm{sin}\left(\theta \right)$, as shown in Eq. (6). The $\mathrm{sin}(\theta )$ term is a result of integration in spherical coordinates. The average mass-to-charge $\overline{\zeta }$ for each angle is based on the time-of-flight data collected at that angle. The current fraction is calculated by normalizing the magnitude of the collector current ${I}_{C}\left(\theta \right)$ to find ${f}_{C}(\theta )$ such that the sum of ${f}_{C}\left(\theta \right)\mathrm{sin}\left(\theta \right)$ over all beam angles equals one.

5 $$\dot{m}={I}_{B}\sum_{\theta }{f}_{C}\left(\theta \right)\overline{\zeta }\left(\theta \right)\mathrm{sin}\left(\theta \right)$$

Here, we computed the mass flow rate using a similar method, replacing the summation in Eq. (6) with an appropriate integral. The current fraction ${f}_{C}(\theta )$ and the average mass-to-charge $\overline{\zeta }(\theta )$ were fitted with continuous functions, and the current fraction was normalized such that the integral of ${f}_{C}\left(\theta \right)\mathrm{sin}\left(\theta \right)$ over the beam equals one (Eq. (6)). The mass flow rate was then calculated by Eq. (7). Note that Eq. (6) and Eq. (7) assume axisymmetry about the center of the current distribution. Eq. (7) is used throughout this work to calculate the propellant mass flow rate from time-of-flight data.

6 $${\int }_{0}^{\pi /2 }{f}_{C}\left(\theta \right)\mathrm{sin}\left(\theta \right)d\theta =1$$

7 $$\dot{m}={I}_{B}{\int }_{0}^{\pi /2}{f}_{C}\left(\theta \right) \overline{\zeta }\left(\theta \right)\mathrm{sin}\left(\theta \right)d\theta$$

## Methodology

### UIUC Retarding Potential / Time-of-Flight Mass Spectrometer (RP/ToF-MS)

**Fig. 2:**
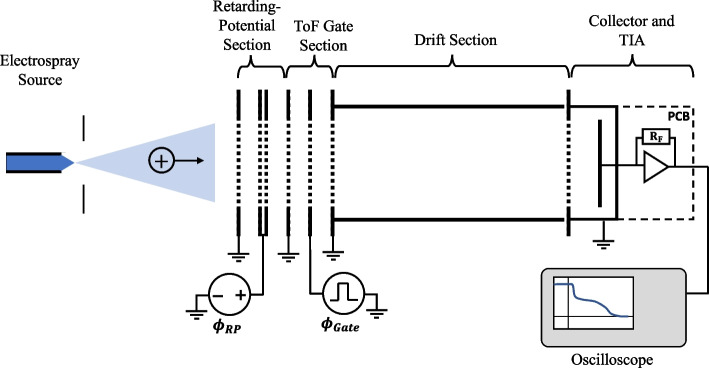
Retarding potential time-of-flight mass spectrometer (RP/ToF-MS) developed at the University of Illinois

The RP/ToF-MS used in this work, shown in Fig. 2, is an extension of the ToF-MS design detailed in [19]. It consists of a retarding potential analyzer coupled to a time-of-flight mass spectrometer. The beam from the electrospray source enters the instrument and passes through the retarding potential section, which blocks species with potentials below the retarding grid potential. After the retarding potential section is the time-of-flight gate, which is used to periodically interrupt the beam. After the gate there is a drift section followed by the collector and transimpedance amplifier (TIA), which converts the collector current to a voltage signal that is output to an oscilloscope. The retarding potential and time-of-flight sections are coupled together in a linear design, like in Lozano’s early work [12]. In our design the retarding potential section is placed before the gate rather than after, so that the charged particles drift between the gate and the collector without changing speed. The linear design used here is considerably less complicated than the orthogonal design used in [16, 17]. As a result, it is much simpler to estimate the instrument’s transparency and the expected collector current. For this work, the collector is a disc with a 19 mm diameter located 167 mm from the electrospray source. The collector subtends a cone with a half angle of 3.3°, which corresponds to a solid angle of 0.1 steradians. Seven mesh screens are used, each with an optical transparency of 88%, giving an overall optical transparency of 41%.

The retarding potential analyzer and the electrostatic gate are formed by five plates separated by 2.5 mm spacers. Each plate has an aperture covered by conductive mesh. The first, third, and fifth plates are held at ground potential. The desired retarding potential is applied to the second plate, which has mesh grids on both sides to provide a more consistent retarding potential across the grid. The fourth plate acts as the electrostatic gate. The gate potential is quickly changed between ground and high voltage using a fast switch to interrupt the electrospray beam. The gate is followed by a drift tube with an inner diameter of 22.9 mm, then by the collector. The collector consists of a 19 mm copper disk surrounded by a grounded shell. The entrance of the collector is covered by a plate with a 22.9 mm aperture covered with conductive mesh. The collector plate is soldered directly to an SMA-style coaxial adapter, which provides an electrical connection between the collector and the transimpedance amplifier via a rigid coaxial connector. The distance between the electrostatic gate and the collector plate (i.e., the flight length, $L$) is 116 mm. The total distance between the electrospray source and the collector plate is 167 mm. Kimball Physics eV parts were used to construct the ion optics (plates, spacers, tubes, etc.), which were spot welded as needed using nickel shim. All grids were made by covering a plate aperture with 90.1 LPI nickel mesh with 88% transparency (MN20, Precision Eforming LLC.).

**Fig. 3:**
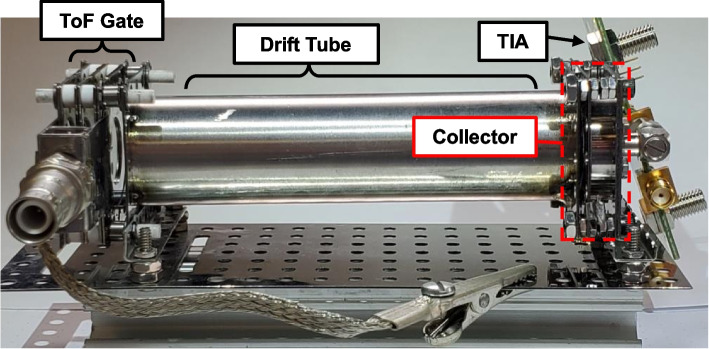
Time-of-Flight Mass Spectrometer used in this work. Shown without the optional retarding potential section

The instrument used in this work is shown in Fig. 3. Note that Fig. 3 does not show the retarding potential analyzer, which is mounted directly to the electrostatic gate for RP/ToF-MS measurements. The instrument is operated in a vacuum chamber with a base pressure of about 10^-6^ Torr. The transimpedance amplifier is located within the vacuum chamber, mounted directly to the collector via a short, rigid coaxial connector. Directly coupling the TIA to the collector has two important advantages. First, for a given bandwidth, reducing the total capacitance between the TIA input and ground allows a larger transimpedance gain (${R}_{F}$) to be used. Since signal-to-noise ratio is roughly proportional to $\sqrt{{R}_{F}}$, this improves the maximum signal-to-noise ratio for collector current measurements. Second, directly coupling the TIA to the collector reduces susceptibility to electromagnetic interference since the cable length between the TIA and the collector is minimized and a well-shielded rigid coaxial connector is used.

Two TIAs were used in this work, each based on the OPA858 op-amp (Texas Instruments), one with ${R}_{F}\approx 1 \mathrm{M\Omega }$ and another with ${R}_{F}=4.8 \mathrm{M\Omega }$. The 10% to 90% rise times were approximately 70 ns and 200 ns, respectively. Note that the feedback resistor for the $4.8 \mathrm{M\Omega }$ TIA is actually three resistors in series, each size 0402. This was done to reduce the parasitic capacitance between the inverting input and the output of the op amp, which results in a faster rise time. In this case, no feedback capacitor is used. Instead, the parasitic capacitance of the feedback resistors acts as a feedback capacitor. When paired with a low input capacitance, estimated to be about 10 pF in this work, this parasitic capacitance is sufficient to keep the TIA stable. More information about TIA stability and fabrication can be found in [19]. The time-of-flight gate was opened and closed by a high-voltage MOSFET switch (Behlke 91-01-HB-C). The 10% to 90% rise/fall time of the gate was less than 200 ns for all data presented here. The majority of this work was carried out using the 4.8 $\mathrm{M\Omega }$ TIA and a gate rise time of 190 ns. For this combination, the noise induced by the gate is equivalent to ~19 nA peak, settling to < 0.5 nA within 800 ns. Although this noise has largely subsided before a useful time-of-flight signal needs to be measured ($t>2 \mu s$), the noise may still be comparable in magnitude to RP/ToF-MS signal levels, which range from ~30 pA to ~400 pA in this work. However, the ‘background noise’ induced by the gate is automatically removed when the time-of-flight signal at ${\phi }_{2}$ is subtracted from the signal at ${\phi }_{1}$ to compute the RP/ToF-MS signal. The same correction can be applied to standard ToF-MS data by recording a ‘background’ signal with no beam present and subtracting it from the measured time-of-flight signals. The output signal from the TIA was recorded using an oscilloscope (Tektronix DPO 2024) with the bandwidth limited to 20 MHz. Note that the DPO 2024 has a waveform averaging feature that we used to significantly improve the signal-to-noise ratio of our measurements.

### Electrospray source

**Fig. 4:**
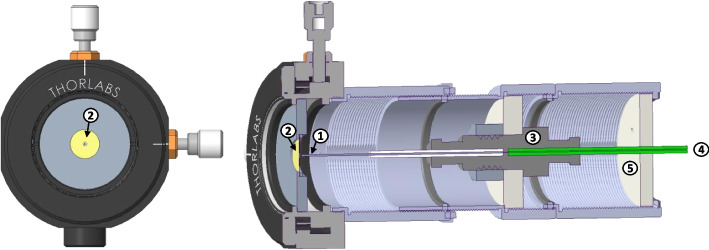
Capillary electrospray source. (1) Emitter, (2) Extractor, (3) Union, (4) Feed Tube, (5) Endcap

The electrospray source used in this work is shown in Fig. 4. It consists of a capillary emitter with an inner diameter of 50 µm (New Objective, TaperTip) enclosed within a grounded lens tube (Thorlabs SM Series) which shields the capillary from secondary species produced by the electrospray beam impacting the vacuum chamber. The lens tube is attached to an alignment mount (Thorlabs LM1XY) which also holds the extractor, allowing the two to be precisely aligned. Propellant is supplied through a fused silica tube with a 100 µm inner diameter. The emitter potential is applied to a stainless-steel union (Valco ZU1XC), which connects the feed tube to the emitter. The high voltage and propellant connections pass through an insulating bulkhead at the rear of the lens tube which allows the emitter to be fully enclosed while still making the necessary connections. The source is mounted on a rotation stage, allowing the beam properties to be studied as a function of angle. For all experiments in this work, the emitter potential was ${\phi }_{E}=+1500 V$, the extractor was grounded, the gap between the emitter tip and the extractor was 500 µm, and the extractor aperture diameter was 1 mm. The time-of-flight instrument used in this work is capable of measuring the mass spectra of positively charged or negatively charged beams. However, the majority of available literature data for EMI-Im capillary electrosprays are for positive emission mode (e.g., [8, 9, 16]), so only positive emission data are presented here.

The propellant reservoir is located in an auxiliary vacuum chamber, and its flow rate is controlled by changing the reservoir pressure. The propellant is conditioned by drying under vacuum to remove volatiles [20]. To obtain direct flow rate measurements, an air bubble is seeded into the propellant feed tubing and its motion is recorded with a microscope and camera. The flow rate is calculated from the velocity of the bubble and the diameter of the tubing. We found that seeding multiple bubbles led to inconsistent flow measurements, possibly due to the accumulation of gas downstream of the measurement section. All direct flow rate measurements reported here were taken using a single seeded bubble.

### Data processing techniques

**Fig. 5:**
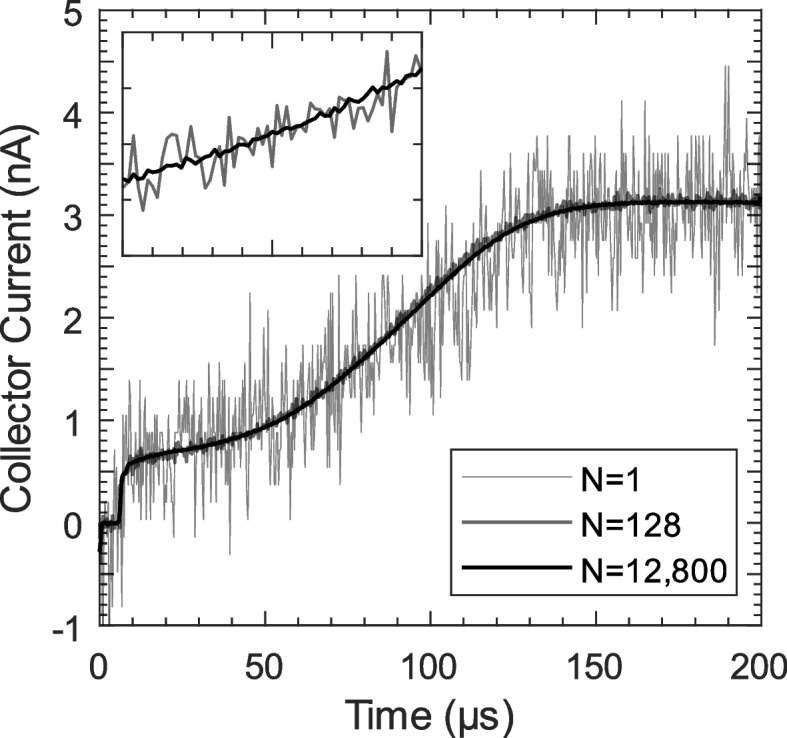
Time-of-Flight curves where the gate opens at ${\varvec{t}}=0$. N denotes the number of ToF signals averaged to obtain the corresponding curve. The inset shows the N=128 and N=12,800 curves in greater detail

Time-of-flight measurements for single-emitter electrospray sources are difficult due to low beam currents (typically ~200 nA to ~500 nA) and the need for high-speed measurements, resulting in a low signal-to-noise ratio. Lyne et al. discuss methods for improving signal-to-noise, including transimpedance amplifier design optimization, shielding components from electromagnetic interference, and using signal averaging [19]. In this work, the time-of-flight signal was acquired by an oscilloscope and transferred to a PC for storage and analysis. In practice, multiple time-of-flight signals are averaged together for each measurement in order to increase the signal-to-noise ratio. Figure 5 shows time-of-flight curves where N denotes the number of signals averaged to obtain the curve.

**Fig. 6:**
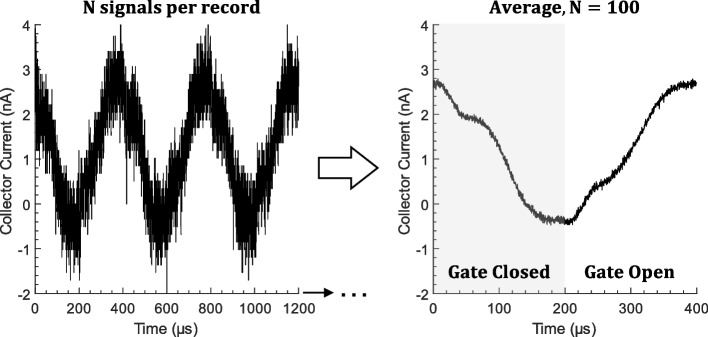
Fast averaging technique used to reduce noise in time-of-flight measurements. Multiple ToF signals are captured in a single waveform record (left). Then, those signals are averaged together to produce a low-noise ToF signal (right). In this work, this technique was used to capture and average up to 842 ToF signals per second

Figure 5 clearly shows that the quality of time-of-flight data increases with the number of signals averaged. Consequently, we developed a technique for quickly acquiring and averaging ToF signals, which is depicted in Fig. 6. This technique works by capturing multiple time-of-flight signals in a single waveform acquisition (Fig. 6 left). After the waveform is acquired, it is split into segments, each of which corresponds to a single ToF signal. Those segments are then averaged together to yield a low-noise ToF signal (Fig. 6 right). In the example shown in Fig. 6, the left waveform contains 125,000 samples and 100 time-of-flight signals. Note that each ToF signal consists of 200 µs with the gate closed, followed by 200 µs with the gate open. After averaging, the resulting waveform contains 1250 samples and a single time-of-flight signal spanning from $t=0$ to $t=400 \mu s$. Note that the gate is controlled by a square wave with a period of 400 µs. The gate switching frequency was chosen so that the time-of-flight signal reaches steady state before the next gate switching event.

ToF signals can be quickly acquired and averaged by combining the technique shown in Fig. 6 with the waveform averaging feature provided by many oscilloscopes, which acquires and averages multiple waveform records. Using a Tektronix DPO 2024 oscilloscope with a record length of 125,000 samples and a timebase of 4 ms, we captured and averaged ToF events at a rate of 235, 543, 681, 780, and 842 ToF events per second with the waveform averaging feature set to 16, 64, 128, 256, and 512 waveforms, respectively. For example, at a setting of 128 waveforms per average, 12,800 ToF events can be captured and averaged in less than 20 seconds. While the rate of data capture will depend on the specific equipment used, the techniques described here can improve the data capture rate for oscilloscopes with appropriate features and sufficient waveform record length.

**Fig. 7:**
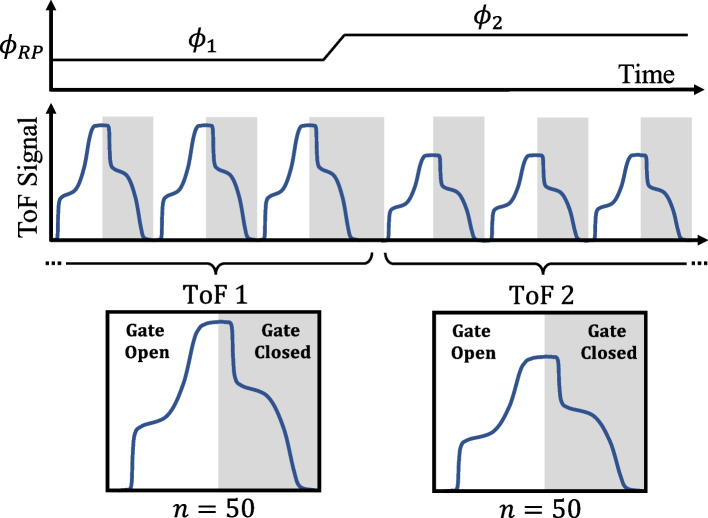
Fast averaging method for retarding potential time-of-flight (RP/ToF-MS) measurements. The first half of the waveform is the ToF signal at ${{\varvec{\phi}}}_{1}$ and the second half is at ${{\varvec{\phi}}}_{2}$. Their difference, ToF 1 – ToF 2, is the time-of-flight signal associated with the range of potentials ${\varvec{\phi}}$ where ${{\varvec{\phi}}}_{1}<{\varvec{\phi}}<{{\varvec{\phi}}}_{2}$

The fast averaging method used for conventional time-of-flight measurements (Fig. 6) was adapted slightly for tandem retarding potential time-of-flight measurements, as shown in Fig. 7. For RP/ToF, each waveform record is split into two halves. During the first half of the waveform record, the potential ${\phi }_{1}$ is applied to the retarding grid. After 50 ToF signals are captured, the retarding potential is increased to ${\phi }_{2}$ and another 50 signals are captured. The signals from the first half are averaged to yield ToF1 and the signals from the second half are averaged to yield ToF2. Then the RP/ToF signal is calculated by subtracting ToF2 from ToF1 (e.g., Fig. 1).

## Results

### Propellant flow rate measurement using time-of-flight

Time-of-flight mass spectrometry is a useful tool for indirectly measuring the thrust and propellant flow rate of electrospray thrusters. ToF-based thrust measurements have been reported accurate to within about 15% of direct thrust measurements for capillary [8] and non-capillary [1] electrospray sources. ToF-based mass flow measurements are more variable, with reported errors ranging from a few percent for capillary sources [10, 11] to over 60% for porous electrospray sources [2]. Furthermore, correlations between emitter current and propellant flow rate for capillary electrosprays of EMI-Im are highly variable, with reported values varying by more than ±30% across the literature [9–11, 16].

**Fig. 8:**
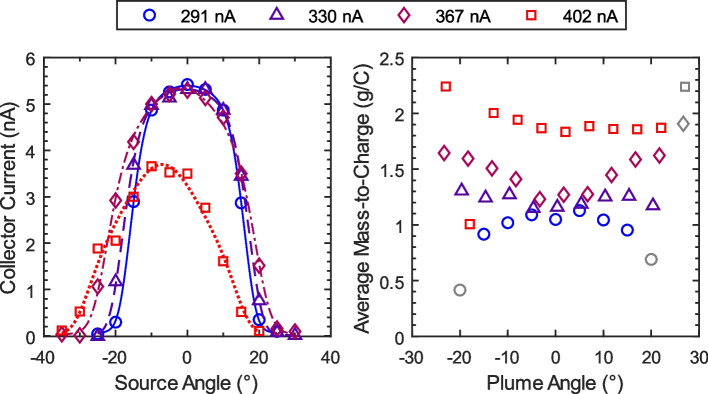
(Left) ToF collector current vs. source angle. (Right) Average mass-to-charge ($\overline{{\varvec{\zeta}} }$) vs. angle from plume center. Gray markers denote data where ${{\varvec{I}}}_{{\varvec{C}}}<0.5\boldsymbol{ }{\varvec{n}}{\varvec{A}}$

Because of these uncertainties, we investigated the accuracy of ToF-MS based flow rate measurements for capillary EMI-Im electrosprays. At each of four flow rates, direct measurements were taken by seeding an air bubble into the propellant feed line and recording its movement with a microscope and camera, then calculating the flow rate from the tubing diameter and bubble velocity. Time-of-flight data were taken as a function of angle in 5° increments spanning the width of the measurable beam. The methods described in Section 2.3 were used to calculate the propellant mass flow rate from these angle-resolved data. The beam current profile was inferred from the collector current data, ${I}_{C}=f(\theta )$, as shown on the left in Fig. 8. The current data (markers) were fit with a super-gaussian distribution (lines) (as described in [21]), which is an excellent fit for the data at the lowest flow rate, where the center of the current distribution is at the geometric center of the plume. As the beam current increases, the current distribution becomes wider, and the plume begins to tilt off axis ($\theta =0.0^\circ ,0.4^\circ ,1.7^\circ ,7.1^\circ$).

The right side of Fig. 8 shows the average mass-to-charge calculated from time-of-flight data at each angle. Note that the x-axis has been changed to ‘plume angle’, which denotes the angle from the center of the current distribution, rather than the geometric angle that the source is turned to. At the lowest beam current, ${I}_{B}=291 nA$, the average mass-to-charge ($\overline{\zeta }$) decreases as beam angle increases. However, as the beam current increases this trend inverts and the beam edges have a higher average mass-to-charge than the center.

**Fig. 9:**
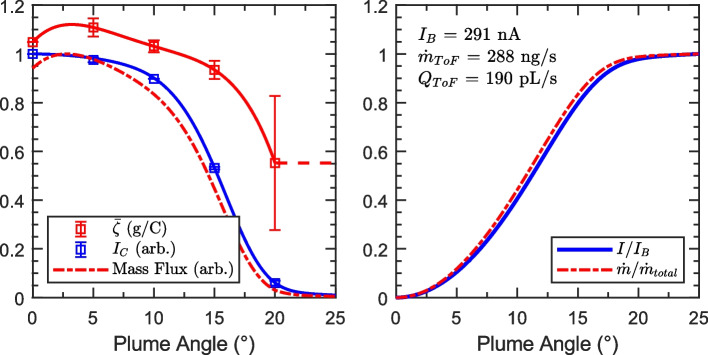
Plume properties for ${I}_{B}=291 nA$. (Left) Current density profile ${I}_{C}$ and average mass-to-charge $\overline{\zeta }$ vs. angle from plume center. (Right) Cumulative distributions of current and mass fractions ($I/{I}_{B}$ and $\dot{m}/{\dot{m}}_{total}$, respectively) vs. included angle

The beam current profile and average mass-to-charge were used to calculate the mass flow rate using Eq. (7). First, the data were fit and assumed to be axisymmetric about the center of the current distribution. The current and average mass-to-charge data were fit with super-gaussian and spline fits, respectively. Figure 9 (Left) shows the fitted distributions for ${I}_{B}=291 nA$. Data for negative plume angles was reflected over $\theta =0^\circ$, then the values for positive and negative angles were averaged and plotted. The error bars on those data markers indicate the spread between those values. The fitted data (solid lines) were used to compute the mass flux profile (dash-dot line). The dashed segment of the average mass-to-charge fit indicates that the uncertainty in $\overline{\zeta }$ is large for the time-of-flight data collected at these angles, so the average mass-to-charge is assumed to be equal to the highest angle where reliable data are available (20° in this case). The right side of Fig. 9 shows the cumulative distributions of current and mass flow rate, which indicate that 80% of the beam current and mass flow fall between 5° and 17° off-center. Nearly 99% of the plume mass flow occurs between 0° and 20° plume angle. Consequently, the uncertainty in average mass-to-charge for plume angles greater than 20° is not a significant source of error in the calculation of mass flow rate.

**Fig. 10:**
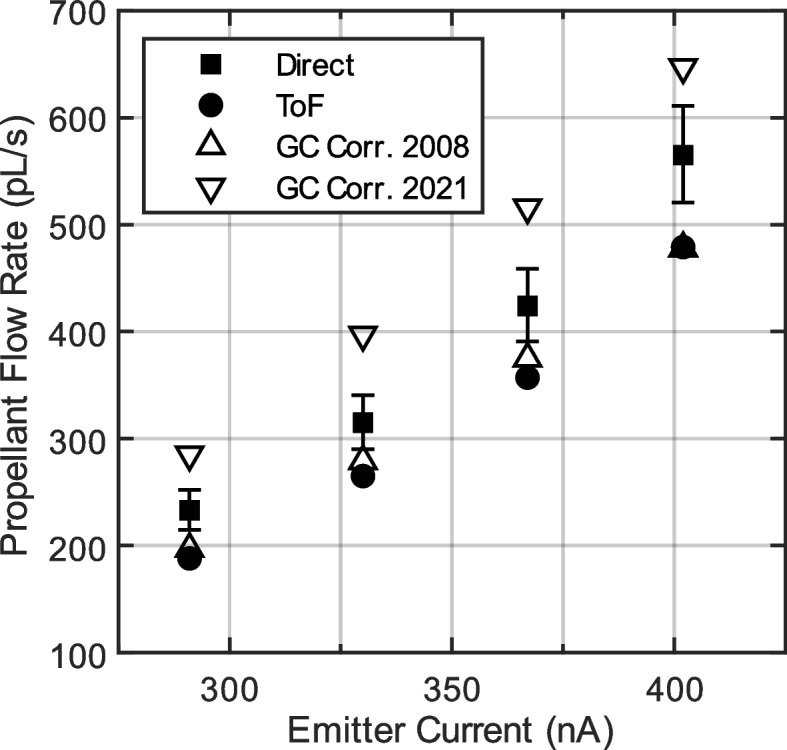
Propellant flow rate as a function of emitter current. The triangle markers represent the flow rate inferred from the emitter current using correlations from the literature [9, 16].

The distributions of $\overline{\zeta }$ and ${I}_{C}$, shown in Fig. 9 for ${I}_{B}=291 nA$, were measured at four different propellant feed pressures and used to calculate the propellant volumetric flow rate (${Q}_{ToF}$) for each. Figure 10 compares those data to simultaneous direct measurements taken by the bubble tracking method. Figure 10 also shows the flow rate inferred from published $Q=f({I}_{B})$ correlations [9, 16]. Using the direct flow rate measurements as a benchmark, the error in ${Q}_{ToF}$ changes from -18% to -15% as beam current rises from ${I}_{B}=291 nA$ to ${I}_{B}=402 nA$. Error bars on the direct measurements result from a 4% tolerance in the inner diameter of the propellant feed tubing.

As discussed in section 2.1, a potential distribution must be assumed to calculate mass-to-charge from time-of-flight data. According to Eq. (2), error in assumed potential ${\phi }_{RP}$ leads to a proportional error in the computed mass-to-charge. For nearly monoenergetic beams the error in potential is small, and the beam potential can be approximated as constant. However, for beams with a large energy spread, assuming a constant beam potential distorts the measured mass-to-charge distribution and leads to error in the measured flow rate. Recent studies of EMI-Im capillary electrosprays using tandem retarding potential analysis and time-of-flight mass spectrometry have found a linear correlation between average mass-to-charge and retarding potential [16, 17]. Using the process described in section 2.1, these potential distributions can be expressed in terms of flight time (${\phi }_{RP}=f\left(\overline{\zeta }\right)\to {\phi }_{RP}=f(t)$) so that they can be used to calculate propellant flow rate by Eq. (7). For example, Gamero-Castaño et al. found linear potential distributions (i.e., slopes and y-offsets) for four different EMI-Im beam currents [16]. From those data, the slopes and y-offsets were tabulated as a function of beam current. Linear interpolation was used to find the appropriate slope and y-offset for the beam currents used in this study.

### Tandem Retarding Potential and Time-of-Flight Methods (RP/ToF-MS)

The strategy of applying a known potential distribution to the analysis of ToF-MS data is made possible by the availability of retarding potential data in the literature. While these data are available for EMI-Im, the potential distribution of the beam is not generally known in advance. Furthermore, using an *average* potential contributes to error in the computed mass-to-charge distribution for beams with large potential spreads, such as capillary electrosprays. A more accurate method for determining the mass-to-charge distribution, thrust, and propellant flow rate is to use an energy analyzer in tandem with ToF-MS. One such method, known as retarding potential time-of-flight mass spectrometry (RP/ToF-MS), uses a retarding potential analyzer to filter the beam potentials that are allowed into the mass spectrometer. Thus, one can measure the ToF-MS signal corresponding to a known, narrow range of retarding potentials. That signal can then be analyzed assuming a constant beam potential without introducing significant error. By sweeping over the range of retarding potentials present in the beam, a composite mass-to-charge spectrum can be obtained without prior knowledge of the beam potential distribution.

**Fig. 11:**
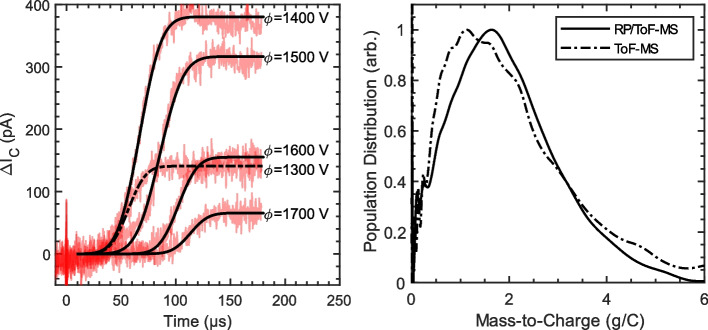
(Left) RP/ToF-MS signals and error function fits in the time domain. (Right) Droplet mass-to-charge population distribution. The solid line shows a RP/ToF-MS composite spectrum, which makes no assumptions about the beam’s energy distribution. The dash-dot line is calculated from conventional ToF-MS data by assuming that potential is a function of mass-to-charge, ${\varvec{\phi}}={\varvec{f}}(\overline{{\varvec{\zeta}} })$.

The left side of Fig. 11 shows RP/ToF-MS signals measured in the plume of an EMI-Im capillary electrospray at ${I}_{B}=285 nA$. To obtain these curves, time-of-flight signals were measured for two retarding potentials spaced 25 V apart (${\phi }_{2}-{\phi }_{1}=25 V$). The difference between those signals gives the time-of-flight curve associated with particles with retarding potentials ${\phi }_{1}<{\phi }_{RP}<{\phi }_{2}$. Those RP/ToF-MS data (red) were fit with error functions in the time domain (black), following the procedure used in [16]. RP/ToF-MS data were taken in 50 V increments spanning from ${\phi }_{RP}={\phi }_{E}-500 V$ to ${\phi }_{RP}={\phi }_{E}+350 V$. The right side of Fig. 11 shows the distribution of mass-to-charge among droplets in the beam, sometimes called the *mass spectrum*. The solid black line shows a composite spectrum obtained from the RP/ToF-MS data, which was computed by summing the spectra at each retarding potential. From this composite distribution, we find that the average mass-to-charge in the beam is ${\overline{\zeta }}_{B}=1.81 g/C$. A mass spectrum obtained from conventional ToF-MS data is also shown (dash-dot line). This spectrum was calculated by assuming that the potential depends on mass-to-charge, $\phi =f\left(\overline{\zeta }\right)$, as was done section 4.1. The average mass-to-charge for this spectrum is nearly the same as the RP/ToF-MS composite spectrum (${\overline{\zeta }}_{B}=1.84 g/C$ vs. ${\overline{\zeta }}_{B}=1.81 g/C$, respectively). This means that by assuming an appropriate potential distribution, the average beam mass-to-charge ratio in the beam can be found with good accuracy from conventional ToF-MS data alone.

A review of the ToF-MS literature for electrospray propulsion reveals little consistency in the potential distributions assumed by authors. Many authors assume a flat potential distribution, perhaps because it is the simplest case to analyze. Again, there is strong evidence that retarding potential is linearly correlated with mass-to-charge for capillary electrosprays of EMI-Im [16, 17]. Nonetheless, calculations using a constant potential can yield reasonable results. For our data, assuming that the retarding potential is equal to the emitter potential (${\phi }_{RP}={\phi }_{E}$) results in ${\overline{\zeta }}_{B}=1.60 g/C$, about 12% lower than the value of ${\overline{\zeta }}_{B}=1.81 g/C$ obtained from the RP/ToF-MS composite spectrum. Sometimes a retarding potential below the emitter potential is assumed, for example based on retarding potential measurements. Assuming a constant potential deficit of 250 V for our data (i.e., ${\phi }_{RP}=1250 V$) results in a calculated average mass-to-charge of ${\overline{\zeta }}_{B}=1.33 g/C$, nearly 27% lower than the value indicated by the composite spectrum. These results make it apparent that using retarding potential and time-of-flight in tandem (i.e., RP/ToF-MS) is the most accurate way to determine the true mass spectrum in capillary electrospray beams, although the average mass-to-charge can be approximated with reasonable accuracy by assuming an appropriate linear potential distribution, ${\phi }_{RP}=f(\overline{\zeta })$.

## Discussion

### Accuracy of flow rate measurements using time-of-flight mass spectrometry

As discussed in the previous section, the potential distribution assumed when analyzing ToF-MS data can have a significant impact on the calculation results. Table 1 contains the propellant flow rate computed from time-of-flight data assuming various potential distributions. These include a flat distribution equal to the emitter potential (${\phi }_{RP}={\phi }_{E}$) and a distribution of the form ${\phi }_{RP}={\phi }_{RP}(\overline{\zeta })$. The function ${\phi }_{RP}(\overline{\zeta })$ is based on the experimental data published by Gamero-Castaño et al., using linear interpolation to match our beam currents [16]. Note that the values listed in Table 1 were calculated from angle-resolved time-of-flight data as described in section 2.3, while the average mass-to-charge values in section 4.2 were calculated based on data from the plume centerline. Table 1 also lists the propellant flow rate inferred from the beam current based on two EMI-Im correlations available from the literature [9, 16]. The error in flow rate for the correlations ranges from -15% to +26% for the flow rates tested. Several factors can influence the relationship between emitter current and flow rate, including temperature effects [16] and secondary charge emission effects if the source is not properly shielded [22–24].

**Table 1:** Comparison of propellant flow rates determined by direct measurement, the angle-resolved time-of-flight method (section 2.3), and current-flow rate correlations

Beam Current	Flow Meter	Time-of-Flight						$Q=f({I}_{B})$ Corr.			
-	*-*	${\upphi }_{\mathrm{RP}}=\mathrm{f}(\overline{\upzeta })$		${\upphi }_{\mathrm{RP}}={\upphi }_{\mathrm{E}}$		${\upphi }_{\mathrm{RP}}=\overline{{\phi }_{RP}}$		GC ’21 [16]		GC ’08 [9]	
	Q	Q	Err.	Q	Err.	Q	Err.	Q	Err.	Q	Err.
nA	pL/s	pL/s	%	pL/s	%	pL/s	%	pL/s	%	pL/s	%
291	233	190	-18%	197	-16%	173	-26%	285	+22%	197	-15%
330	315	265	-16%	273	-13%	233	-26%	397	+26%	279	-11%
367	424	357	-16%	358	-16%	296	-30%	516	+22%	375	-11%
402	565	479	-15%	494	-13%	394	-30%	647	+15%	478	-15%

There are many possible reasons that the ToF-MS-based flow rate measurement could underestimate the propellant flow rate. The first factor to consider is the effect of the assumed potential distribution. Recent studies using RP/ToF-MS have shown that average retarding potential in EMI-Im capillary electrospray beams is a linear function of mass-to-charge, i.e., ${\phi }_{RP}=f(\overline{\zeta })$ [16, 17]. Using ${\phi }_{RP}=f(\overline{\zeta })$ fits from Gamero-Castaño et al. [16], we found that ToF-based estimates of propellant flow rate are 18% to 15% lower than direct flow rate measurements using a bubble flow meter. Interestingly, assuming that the retarding potential is simply equal to the emitter potential (${\phi }_{RP}={\phi }_{E}$) reduces the error by 2-3%. To accurately calculate the mass flow rate from ToF data using an average retarding potential, the mass-averaged retarding potential should be used. Unfortunately, calculating the mass-weighted average of retarding potential is not always possible. Rather, the *charge*-weighted average is often used because it can be directly computed from retarding potential analyzer data, which is simple to obtain and widely available in the literature. Interpolating the retarding potential measurements from [9], we find that the average potential deficit is ${\phi }_{E}-\overline{{\phi }_{RP}}=\left\{181, 221, 259, 303\right\} V$ for beam currents ${I}_{B}=\left\{291, 330, 367, 402\right\} nA$, respectively. Using these average retarding potentials (i.e., ${\phi }_{RP}=\overline{{\phi }_{RP}}$), we find that the ToF method underestimates the direct flow rate measurements by 26% to 30%. The cause of this increased error is that $\overline{{\phi }_{RP}}$ is a charge-averaged retarding potential, rather than mass-averaged. Since low mass-to-charge species have the lowest retarding potential (see [16], for example), the average retarding potential weighted by charge is lower than that weighted by mass.

Other factors likely contribute to the error in ToF-based flow rate estimation as well. For example, Fig. 8 shows that that the assumption of beam axisymmetry is poor for high beam currents. However, the error in the ToF flow rate estimate is highest for low beam currents, where the beam is more axisymmetric. Another possibility is that the beam contains a non-negligible fraction of neutrals, which are not detected by the time-of-flight collector. We should note, these supposed neutrals would need to have formed in the acceleration region between the emitter and the extractor in order to cause errors in the time-of-flight data. Neutrals formed by the fragmentation of droplets or ion clusters in the drift region (post acceleration) do not affect the velocity or charge of the parent droplet, so the flight time and signal magnitude are unaffected. The simplest explanation for the discrepancy between flow rates measured using the bubble flow meter and the ToF estimates is that a small leak exists between the propellant feed tube and the emitter, causing some portion of the propellant flow to not reach the emitter tip. However, post-test inspection of the source did not reveal evidence of a leak. In addition, the emitter did not short to ground even after prolonged operation, so we conclude that a leak is an unlikely explanation for the observed error.

One more phenomenon is worth considering in the context of ToF-MS measurements- secondary charge emission. There is considerable evidence, both experimental and computational, that electrospray beams impinging on surfaces can cause charged species emission from the surface [22–24]. Possible secondary species are fragments of the impacting droplet or molecular cluster, electron emission, or sputtering of the surface material. As the electrospray beam bombards the time-of-flight collector, charge emission from the collector surface will affect the measured current. Uchizono et al. have measured the secondary charge yields for a capillary electrospray of EMI-Im as a function of beam potential, beam current, and angle from the beam centerline [23]. They found that significant charge emission occurs at beam potentials as low as 1 kV, and that the positive and negative charge yields are not necessarily the same. Using their data for a 260 nA beam at a beam potential of 1.4 kV, we expect our net secondary charge yield to be about +6% on the beam centerline (${\gamma }_{+}=0.126, {\gamma }_{-}=0.063$). Secondary charge emission at higher beam angles is, however, more significant. Their data indicate for the same beam, net charge yield rises to 10.5% at an off-axis angle of 15°, to 25.7% at 17.5°, and to over 100% at 20°. Thus, we can expect time-of-flight data taken at high angles to be significantly affected by secondary charge emission. Furthermore, secondary charge yield is likely to be a function of mass-to-charge, as suggested by the change in yield between the inner portion of the beam (< 15°), which contains a significant ion population, and the outer portion of the beam (> 15°), which does not. Furthermore, the sign of this effect is opposite for positive and negative yields, greatly increasing the net charge yield at high angles. The influence of mass-to-charge on secondary charge yield is important in the context of time-of-flight experiments because of its differential effect on the signal intensity of various mass-to-charge populations, distorting the measured population distribution. In this work, no corrections for secondary charge emission were made, and the time-of-flight collector was kept at ground potential and surrounded by a grounded shell. Future efforts may seek to use RP/ToF-MS to study the relationship between charge yield and mass-to-charge. For example, measurements could be made with the collector at ground, positive, and negative potentials, as was done in [23]. These three RP/ToF-MS signals could then be used to calculate the secondary charge yields (${\gamma }_{+}$ and ${\gamma }_{-}$) as a function of mass-to-charge.

### Jet breakup parameters

**Fig. 12:**
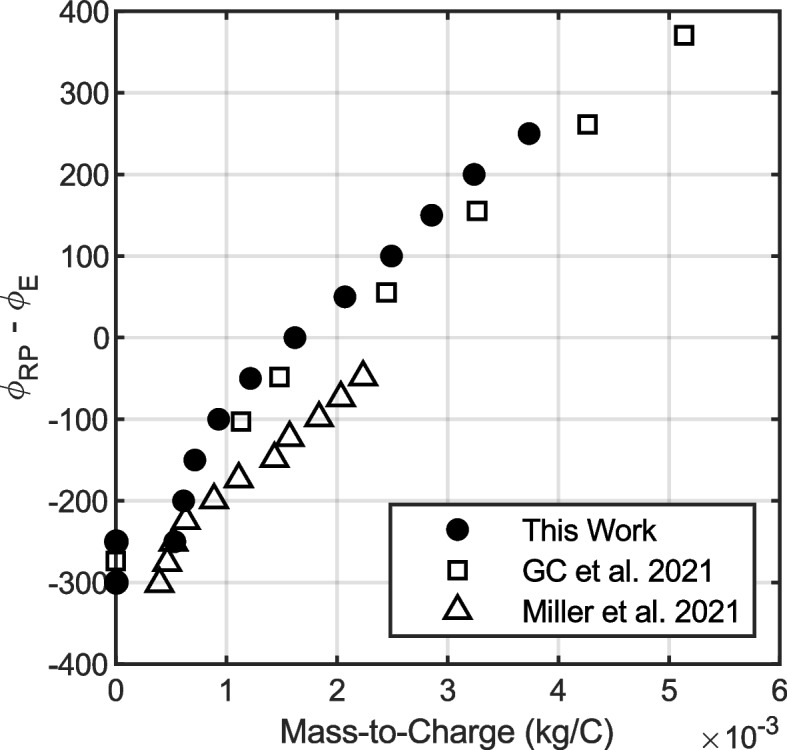
Excess potential (${{\varvec{\phi}}}_{{\varvec{R}}{\varvec{P}}}-{{\varvec{\phi}}}_{{\varvec{E}}}$) as a function of average mass-to-charge.

One application of RP/ToF-MS is to measure the potential distribution in a beam as a function of mass-to-charge. For example, Miller et al. and Gamero-Castaño et al. have each used orthogonal RP/ToF-MS instruments to show that there is a linear correlation between retarding potential and average mass-to-charge in EMI-Im capillary electrosprays [16, 17]. Figure 12 shows those data, using “excess potential” ${\phi }_{RP}-{\phi }_{E}$ on the y-axis to compare data sets taken at different emitter potentials. The Gamero-Castaño et al. data are for a beam current of 300 nA and a flow rate of 297 pL/s. The Miller et al. data are for a flow rate of 280 pL/s and a beam current of 293 nA. Data collected using the linear RP/ToF-MS described in this work are plotted for a beam current of 285 nA and a flow rate of 221 pL/s. Those data are the average mass-to-charge measured at a given retarding potential, determined by fitting the raw RP/ToF-MS data with error functions in the time domain, as described in [16]. For our data, fits with r-squared values lower than 0.4 were excluded from Fig. 12 and from jet breakup calculations. The data shown in Fig. 12 for this work were calculated from the same data set used to find the composite spectrum shown in Fig. 11.

Figure 12 shows that a significant portion of the beam has a retarding potential greater than the emitter potential. To understand this phenomenon, we must consider the physics of capillary electrospray emission. At the tip of the capillary emitter is the liquid meniscus, which forms a Taylor cone in the applied electric field. For capillaries spraying in the cone-jet regime, a charged jet emanates from the tip of this cone with mass-to-charge ${\zeta }_{j}$. This jet accelerates through the electric field and eventually breaks up after traveling through a potential drop of ${\Delta \phi }_{j}$ and reaching velocity ${v}_{j}$. The jet breaks up into droplets with a distribution of mass-to-charge, which accelerate through the remaining potential drop ${\phi }_{E}-\Delta {\phi }_{j}$. The resulting potential distribution is given by Eq. (8). Subtracting the emitter potential from each side, we can rewrite this distribution in terms of the “excess” potential, ${\phi }_{RP}-{\phi }_{E}$, as shown in Eq. (9).

8 $${\phi }_{RP}=\frac{{v}_{j}^{2}}{2}\zeta +\left({\phi }_{E}-\Delta {\phi }_{j}\right)$$

9 $${\phi }_{RP}-{\phi }_{E}=\frac{{v}_{j}^{2}}{2}\zeta -\Delta {\phi }_{j}$$

Conservation of energy dictates that the excess potential be less than or equal to zero for a mass-to-charge ratio equal to the jet mass-to-charge, i.e., when Eq. (9) is evaluated with $\zeta ={\zeta }_{j}$. For our data in Fig. 12 the jet mass-to-charge is ${\zeta }_{j}=1.18 g/C$, which corresponds to an excess potential of -57 V. This excess potential is indeed negative, which confirms that our observation that some droplets have retarding potentials above the emitter potential does not violate conservation of energy. The magnitude of the excess potential at the jet mass-to-charge ratio (i.e., -57 V) gives a measure of losses associated with electrospray emission, for example due to ohmic dissipation. Eq. (9) implies that a droplet with mass-to-charge greater than the jet ($\zeta >{\zeta }_{j}$) can have retarding potentials exceeding the emitter potential, while droplets with $\zeta <{\zeta }_{j}$ will have retarding potentials below the emitter potential. The jet breakup parameters are calculated by applying a linear fit to the data in Fig. 12 and setting the slope equal to ${v}_{j}^{2}/2$ and the y-intercept equal to $-\Delta {\phi }_{j}$. Note that low mass-to-charge droplets are often excluded from this fit because they do not typically conform to the linear trend, likely due to ion field evaporation and Rayleigh fission affecting the most highly charged (lowest $\zeta$) droplets [16]. Applying this methodology, we calculate a jet breakup velocity of ${v}_{j}=491 m/s$ and a jet potential drop of $\Delta {\phi }_{j}=197 V$. These values are a close match to literature data for similar electrosprays. Gamero-Castaño et al. found a jet velocity of ${v}_{j}=481 m/s$ and jet potential drop of $\Delta {\phi }_{j}=225 V$ for a 300 nA EMI-Im beam at a flow rate of 297 pL/s. Similarly, Miller et al. found a jet velocity of ${v}_{j}=466 m/s$ and jet potential drop of $\Delta {\phi }_{j}=297 V$ for EMI-Im at 280 pL/s and a beam current of 293 nA. Each of these jet breakup parameters were calculated from a linear fit of high mass-to-charge droplets $(\zeta >1 g/C)$. These results are summarized in Table 2.

**Table 2:** Summary of jet breakup parameters for EMI-Im capillary electrosprays. The jet velocity (${{\varvec{v}}}_{{\varvec{j}}}$) and jet potential drop (${\varvec{\Delta}}{{\varvec{\phi}}}_{{\varvec{j}}}$) were determined from the RP/ToF-MS data shown in Fig. 12

Authors	${v}_{j}$(m/s)	$\Delta {\phi }_{j}$(V)	${I}_{B}$(nA)	$Q$(pL/s)
Lyne et al. [This work]	491	197	285	221 (est.)
Gamero-Castaño et al. [16]	481	225	300	297 (est.)
Miller et al. [17]	466	297	293	280 (est.)

Table 2 also contains the beam current ${I}_{B}$ and propellant flow rate $Q$, which are important similarity parameters for comparing electrosprays. When one wishes to compare ‘similar’ capillary electrospray results for a given propellant, similarity can be assessed on the basis of flow rate and beam current. Note that all flow rates reported in Table 2 are estimates because the flow rate was not directly measured at the same time as RP/ToF-MS data was acquired. Each of the three studies used a bubble flow meter to measure flow rate. Gamero-Castaño et al. used the flow meter to measure flow rate as a function of feed pressure, verifying that Poiseuille flow accurately predicted the flow rate. Flow rate values reported in their paper are inferred from the applied feed pressure. There is not sufficient detail given in the paper to assess confidence in this reported flow rate. Similarly, Miller et al. used a bubble flow meter to measure the flow rate as a function of feed pressure at “a minimum of four discrete flow rates.” The flow rate data they report were determined by extrapolating this relationship for each feed pressure. Again, there is insufficient detail to assess confidence in their flow rate data. In this work, bubble flow meter data were collected at four discrete flow rates, and the emitter current for each was recorded. The flow rate value listed for this work in Table 2 was determined by extrapolating this relationship to match the emitter current (i.e., 285 nA). Although the flow rate was found by extrapolation, the query point is very close to the nearest direct measurement (285 nA vs. 291 nA), so we have a high confidence in the flow rate we report in Table 2. It is common across the capillary electrospray literature to rely on Poiseuille flow to indirectly ‘measure’ propellant flow rate by measuring the applied feed pressure. While this approach has an analytical basis, we believe that it is less reliable than inferring flow rate from the flow rate – emitter current relationship. Our reasoning is that partial or full clogging of the propellant feed line and the emitter is common, causing the flow rate to decrease despite a constant feed pressure. In this case, the emitter current will decrease with the flow rate, making the emitter current a more robust proxy for the flow rate than feed pressure. We should note, however, that the emitter current – flow rate relationship is sensitive to the temperature at the emission site, so care should be used when inferring flow rate from emitter current data.

### Summary of RP/ToF-MS Instruments for Electrospray Propulsion

The previous section compared three RP/ToF-MS instruments from the electrospray propulsion literature, demonstrating that they have similar capabilities. In this section, we discuss their differences, and we attempt to help the reader choose an appropriate technology for their experiment. Our first point is to note that the instruments discussed in this paper are designed to study charged particle beams, namely those produced by electrospray sources. In addition to the capabilities demonstrated here, mass spectrometers used to analyze biological or chemical samples must include an ionization source and must accelerate those ions before they enter the time-of-flight section. Ion acceleration is often accomplished using orthogonal ToF (e.g., [13]) though many other ion sources and mass spectrometry technologies are described in the literature. Here, we will confine our discussion to ToF-MS instruments that are intended to study charged particle beams (e.g., electrospray plumes). Our second point is that sensitivity and resolution must be balanced against cost and size for any instrument. High sensitivities and resolutions can be achieved using advanced detectors such as electron multipliers, but they drive up system cost. Similarly, resolution can be improved by increasing the flight distance that ions must travel, causing the instrument to be larger. Which instrument is ‘best’ depends on one’s needs and budget.

Table 3 summarizes some key information about each of the three instruments we’ve discussed. Each uses a different means of filtering the incoming particle energies. This work uses a simple linear retarding potential analyzer, which acts as a high-pass filter for particle energy. That is, particles with potentials greater than the retarding potential enter the ToF-MS. Both of the other instruments use energy filtering techniques that act as a bandpass filter, only allowing particles within a narrow range of energies to enter the ToF-MS. The high-pass filtering method used in this work requires two time-of-flight measurements to be made at different retarding potentials in order to produce a single RP/ToF-MS curve. In contrast, the two instruments using bandpass energy filtering methods can acquire a RP/ToF-MS curve in a single measurement.

**Table 3:** Summary of tandem RP/ToF-MS instruments in the electrospray propulsion literature. Abbreviations: RPA- Retarding Potential Analyzer, ToF- Time-of-Flight, MCP- Multichannel Plate detector

Instrument	Energy Filter	Mass Spec.	Detector Type	Suitable For
Lyne et al. [This work]	Linear RPA	Linear ToF	Metal Plate	Ions and Droplets
Gamero-Castaño et al. [16]	Deflection RPA	Linear ToF	Metal Plate	Droplets
Miller et al. [15, 17]	Orthogonal ToF	Orthogonal ToF	MCP	Ions and Droplets

Next, we can consider the differences in mass spectrometer design. Both this work and Gamero-Castaño et al. use a linear time-of-flight mass spectrometer. However, the collector currents reported here for RP/ToF-MS data are about two orders of magnitude higher than those reported by Gamero-Castaño et al. [16], due to differences in energy filtering method and the solid angle covered by the detector. The exceptionally high gain needed to resolve such small signals leads to an increase in the response time of the amplifier. Furthermore, the single picoamp signals measured by Gamero-Castaño et al. may have led them to prioritize ultra-low input bias current when selecting an amplifier, though they do not report what model of amplifier they used. In contrast, we prioritized selecting the fastest available amplifier (gain bandwidth product of 5500 MHz) for our design, allowing for a high gain while still providing a fast response time. Consequently, their instrument is not capable of resolving ions in the mass spectrum, simply because their flight time is too short to be accurately resolved by the amplifier electronics.

In contrast, Miller et al. use a an orthogonal time-of-flight mass spectrometer for energy filtering and mass spectrometry [15, 17]. Their instrument filters particle energies using a set of parallel plates held at a constant retarding potential. As particles enter the region between the plates, they are slowed down by the plate potential. One plate periodically pulses with a high voltage, ‘pushing’ ions in the region towards the other plate, which has a mesh screen allowing particles to pass through. Only particles with energies slightly above the plate retarding potential are successfully extracted into the orthogonal axis, where they travel a distance before reflecting off the reflectron and then reaching the detector. The instrument described by Miller et al. is the most complex and expensive of the three we’ve discussed, but it also has several distinct advantages. First, the long flight length and ultra-fast response time of their multichannel plate (MCP) detector results in a far better resolution than the instrument in this work or by Gamero-Castaño et al. Resolution for time-of-flight mass spectrometers is given by Eq. (10 [13]. Typically, resolution is measured or calculated for a specific mass-to-charge ratio and accelerating voltage, then reported in terms of the full width at half maximum for that peak in the mass spectrum. The resolution reported by Miller et al. is $R\approx 340$ at a mass-to-charge of 1974 amu/q. We do not have mass spectrum data to directly compare to that figure, so we will estimate resolution from the time response of the electronics, which have a 10% to 90% rise time of 200 ns. Using those numbers and assuming an acceleration potential of 1250 V, we estimate that the instrument presented in this work has a resolution of about 26 at a mass-to-charge ratio of 1974 amu/q, more than an order of magnitude lower than Miller et al.

10 $$R=\frac{m}{\Delta m}=\frac{\zeta }{\Delta \zeta }=\frac{t}{2\Delta t}$$

Perhaps the most significant difference between the instrument presented in this work and the orthogonal ToF presented by Miller et al. is that the latter uses a multichannel plate detector (MCP), which significantly increases the sensitivity of the instrument compared to simply using a metal plate as a detector. When an ion strikes the MCP, it starts a cascade of charge that eventually reaches a charge detector. The charge reaching the detector can be many orders of magnitude higher than the incident charge, thus the signal is amplified and made easier to detect. The high gain of the MCP used by Miller et al. allow them to achieve a much higher sensitivity than the instrument described in this work. For example, fig. 3d in their manuscript shows that they detect ion clusters in the plume as large as one ion bonded with six neutral pairs [17], which is far below the detection limit of our instrument. One must consider the cost-benefit tradeoff when choosing a detector. If very high sensitivity and resolution are a priority, then an MCP or similar technology should be considered. However, if a lower resolution and sensitivity will meet the needs of an experiment, a simple metal plate detector and an appropriately designed transimpedance amplifier provide an inexpensive alternative.

## Conclusion

We have investigated the accuracy of time-of-flight methods for measuring the propellant flow rate for capillary electrospray beams. Using a linear time-of-flight mass spectrometer (ToF-MS), we collected data as a function of angle in the plume of a single capillary emitter spraying the ionic liquid propellant EMI-Im at four flow rates. For each flow rate, a bubble flow meter was used to obtain direct flow rate measurements. These direct measurements were compared to ToF-based measurements for a variety of assumed potential distributions. The flow rates calculated from ToF data were lower than the direct measurements by 13% to 30% (Table 1). We found that assuming a constant retarding potential equal to the emitter potential (${\phi }_{RP}={\phi }_{E}$) resulted in errors of -18% to -15%. Assuming a constant potential equal to the average retarding potential measured by a retarding potential analyzer, ${\phi }_{RP}=\overline{{\phi }_{RPA}}$, resulted in larger errors ranging from -26% to -30%. We also analyzed ToF data assuming a linear correlation between retarding potential and mass-to-charge using data from Gamero-Castaño et al. (${\phi }_{RP}=f(\overline{\zeta })$), which resulted in errors nearly identical to those obtained by assuming a constant retarding potential equal to the emitter potential. Although there is strong evidence to suggest that a linear potential distribution is more realistic, the flow rates calculated assuming ${\phi }_{RP}=f(\overline{\zeta })$ are no more accurate than those calculated by assuming ${\phi }_{RP}={\phi }_{E}$. Consequently, we recommend that experimentalists simply use the emitter potential when calculating the propellant flow rate from time-of-flight data.

Next, a retarding potential analyzer was operated in tandem with the ToF-MS. The tandem RP/ToF-MS instrument was used to measure the mass spectrum at the plume centerline, without making any assumptions about the potential distribution in the plume. This composite spectrum was compared to the spectrum obtained from ToF-MS data (Fig. 11, Right) under the most realistic assumed potential distribution, ${\phi }_{RP}=f(\overline{\zeta })$. The comparison demonstrates that the spectrum obtained from ToF-MS data is distorted, but that the average mass-to-charge only differs from the composite spectrum by 2% at the plume centerline. Unlike the angle-resolved measurements discussed above, assuming a constant retarding potential when analyzing data near the plume center results in a higher error than assuming a linear potential distribution. Comparing the calculated average mass-to-charge to that of the composite spectrum, we found an error of -12% for ${\phi }_{RP}={\phi }_{E}$, -27% for ${\phi }_{RP}={\phi }_{E}-250 V$, and +2% for ${\phi }_{RP}=f(\overline{\zeta })$. One plausible explanation for why the linear potential distribution yields more accurate results near the plume center is that the potential distribution was derived from RP/ToF-MS data collected at the plume center by Gamero-Castaño et al. Thus, it is reasonable to expect that potential distribution to be most accurate near the center of the plume.

Last, we calculated the average mass-to-charge as a function of retarding potential (i.e., $\overline{\zeta }=f({\phi }_{RP})$) from our RP/ToF-MS data. These results were compared with recent literature data obtained from two different orthogonal RP/ToF-MS instruments (Fig. 12). A linear fit of high mass-to-charge droplets (> 1 g/C) was used to calculate the jet breakup velocity and breakup potential. The values computed from our data match published values within 2% for jet velocity and 12% for jet breakup potential. These results indicate that the linear RP/ToF-MS has comparable capabilities to orthogonal instruments, despite its simple design and ease of operation.

In summary, the linear RP/ToF-MS design has significant advantages over more complex orthogonal RP/ToF-MS instruments. The linear design is more versatile, since it can be used in conventional ToF-MS mode by keeping the retarding potential grid grounded. In addition, when operating in RP/ToF-MS mode, the linear design allows direct control over the range of retarding potentials included in a given measurement. This is because the linear RP/ToF-MS signal is calculated from two ToF-MS signals taken at different retarding potentials, $${\phi }_{1}$$ and $${\phi }_{2}$$, thus the range of potentials included in the RP/ToF-MS signal is $${\phi }_{1}<\phi <{\phi }_{2}$$. In contrast, the range of potentials included in measurements by the orthogonal instruments cited in this work is set by the ion optics and cannot be easily changed. The unrestricted adjustment of the potential range included in a scan allows the linear RP/ToF-MS to make measurements with higher resolution (smaller $$\Delta \phi$$) or larger signal (larger $$\Delta \phi$$) as needed. Finally, the linear design is simpler to build and operate, also making it less expensive. For example, the RP/ToF-MS used in this work can be built for approximately $2,500 USD, not including the high voltage power supplies used for the gate and retarding potentials or the oscilloscope used for data acquisition. Using the techniques described here and in our previous paper, [19], ToF-MS and RP/ToF-MS systems are within the reach of researchers with even a modest budget.

**Nomenclature**

*ϕ*_*RP*_ retarding potential (also called stopping potential), Volts

*ϕ*_*j*_ jet breakup potential, Volts

*ζ* mass-to-charge ratio, kilograms per Coulomb

$\overline{\zeta }$ average mass-to-charge, kilograms per Coulomb

*v*_*j*_ jet breakup velocity, meters per second

*T* thrust, Newtons

*t* flight time, seconds

*L* flight length, meters*m ̇* propellant mass flow rate, kilograms per second

*Q* propellant volumetric flow rate, cubic meters per second

*I*_*B*_ electrospray beam current, Amps

*I*_*C*_ time-of-flight collector current, Amps

*I'(t)* time-of-flight collector current scaled by ${I}_{B}$, Amps

ToF time-of-flight (abbreviation)

MS mass spectrometer/spectrometry (abbreviation)

RP retarding potential (abbreviation)

## Supplementary Information

**Additional file 1.**

## Data Availability

The full data sets used to produce the paper figures were uploaded as supplementary material.

## References

[1] Courtney DG, Dandavino S, Shea H (2016). Comparing Direct and Indirect Thrust Measurements from Passively Fed Ionic Electrospray Thrusters. J Propuls Power.

[2] Natisin MR (2021). Efficiency Mechanisms in Porous-Media Electrospray Thrusters. J Propuls Power.

[3] Krejci D, Mier-Hicks F, Thomas R, Haag T, Lozano P (2017). Emission characteristics of passively fed electrospray microthrusters with propellant reservoirs. J Spacecr Rockets.

[4] Legge RS, Lozano PC (2011). Electrospray Propulsion Based on Emitters Microfabricated in Porous Metals. J Propuls Power.

[5] Gassend B, Velásquez-García LF, Akinwande AI, Martínez-Sánchez M (2007). “A fully integrated microfabricated externally wetted electrospray thruster”, AIAA 2007–5182, 43rd AIAA/ASME/SAE/ASEE Joint Propulsion Conference, Cincinnati. OH.

[6] Lozano P, Martínez-Sánchez M (2005). Ionic liquid ion sources: Characterization of externally wetted emitters. J Colloid Interface Sci.

[7] Lozano PC (2006). Energy properties of an EMI-Im ionic liquid ion source. J Phys D Appl Phys.

[8] Gamero-Castaño M (2004). Characterization of a six-emitter colloid thruster using a torsional balance. J Propuls Power.

[9] Gamero-Castaño M (2008). Characterization of the electrosprays of 1-ethyl-3-methylimidazolium bis(trifluoromethylsulfonyl) imide in vacuum. Phys Fluids..

[10] Grustan-Gutierrez E, Gamero-Castaño M (2017). Microfabricated electrospray thruster array with high hydraulic resistance channels. J Propuls Power.

[11] Gamero-Castaño M, Hruby V (2001). Electrospray as a Source of Nanoparticles for Efficient Colloid Thrusters. J Propuls Power.

[12] P. C. Lozano, “Studies on the ion-droplet mixed regime in colloid thrusters,” 2003. http://ssl.mit.edu/publications/theses/PhD-2003-Lozano-TovarPaulo.pdf

[13] Chen L, Wan X, Jin DZ, Tan XH, Huang ZX, Tan GB (2015). A compact time-of-flight mass spectrometer for ion source characterization. Rev Sci Instrum.

[14] Enloe CL, Shell JR (1992). Optimizing the energy resolution of planar retarding potential analyzers. Rev Sci Instrum.

[15] Miller SW, Prince BD, Bemish RJ (2017). Orthogonal time-of-flight mass spectrometry of an ion beam with a broad kinetic energy profile. Rev Sci Instrum..

[16] Gamero-Castaño M, Cisquella-Serra A (2021). Electrosprays of highly conducting liquids: A study of droplet and ion emission based on retarding potential and time-of-flight spectrometry. Phys Rev Fluids.

[17] Miller SW, Ulibarri-Sanchez JR, Prince BD, Bemish RJ (2021). Capillary ionic liquid electrospray: Beam compositional analysis by orthogonal time-of-flight mass spectrometry. J Fluid Mech.

[18] M. Gamero-Castaño, “Electric-Field-Induced Ion Evaporation from Dielectric Liquid,” pp. 1–4, 2002, doi: 10.1103/PhysRevLett.89.147602. 10.1103/PhysRevLett.89.147602 12366074

[19] C. T. Lyne, M. F. Liu, J. L. Rovey, “A Low-Cost Linear Time-of-Flight Mass Spectrometer for Electrospray Propulsion Diagnostics,” IEPC-2022–178, 37th International Electric Propulsion Conference, Cambridge, MA, 2022. http://eplab.ae.illinois.edu/Publications/IEPC-2022-178.pdf 10.1007/s44205-023-00045-y PMC10066156 37016724

[20] C. T. Lyne, J. Rovey, S. Berg, “Drying methods for [Emim]+ based ionic liquid electrospray propellants,” AIAA 2022–0038, AIAA SCITECH Forum, San Diego, CA, 2022. 10.2514/6.2022-0038

[21] Thuppul A, Collins AL, Wright PL, Uchizono NM, Wirz RE (2021). Mass flux and current density distributions of electrospray plumes. J Appl Phys..

[22] Uchizono N. M, Collins A. L, Marrese-Reading C, Arestie S. M, Ziemer J. K, Wirz RE (2021). The role of secondary species emission in vacuum facility effects for electrospray thrusters. J Appl Phys..

[23] Uchizono N. M, Marrese-Reading C, Arestie S. M, Collins A. L, Ziemer J. K, Wirz R. E (2022). Positive and negative secondary species emission behavior for an ionic liquid electrospray. Appl Phys Lett..

[24] M. R. Klosterman, J. L. Rovey, D. A. Levin, and A. Rao, “Ion-induced charge emission from unpolished surfaces bombarded by an [Emim][BF4] electrospray plume,” J. Appl. Phys., vol. 131, no. 24, 2022, 10.1063/5.0119297

