# Supplementary material for: A simple retarding-potential time-of-flight mass spectrometer for electrospray propulsion diagnostics
Source: J Elect Propuls. 2023 Mar 31;2(1):13. doi: 10.1007/s44205-023-00045-y (PMC10066156; doi:10.1007/s44205-023-00045-y)
Supplement: Supplementary file 1 — Additional file 1. [file 44205_2023_45_MOESM1_ESM.zip › Data and MATLAB Code/RP ToF-MS Data/RP ToF Matlab Code (html version)/RP_TOF_Data_Analysis.html]

RP\_TOF\_Data\_Analysis 

## Contents

- File I/O and User-Adjustable Settings
- Process Data
- Fit error functions to high amu/q range droplets
- Finish loading calcResults structure with results
- Plot all data on same time x-axis with error function fits for high m/q range
- Plot all data on same m/q x-axis (kg/C)
- Plot m/q center (center of erf fit) vs Retarding Potential
- Plot: Compare to GC '21 FIG 8 and Miller '21 Excess Potential
- Plot: Calculate jet velocity and breakup potential from my data
- Console output
- Functions

```
%
%   Supplementary materials for "A Simple Retarding-Potential Time-of-Flight
%       Analyzer for Electrospray Propulsion Plume Diagnostics" (2022),
%       Submitted to the Journal of Electric Propulsion
%
%   Date: November 2022
%
%   Code Author: Christopher T. Lyne
%       Email: CLyne2@Illinois.edu
%       Alt:   Christopherlyne@gmail.com
%
%   Manuscript Authors: Christopher T. Lyne, Miron F. Liu, Joshua L. Rovey
%
%   Purpose of code:
%       This code, written to run in Matlab R2021a, is used to analyze the
%       RP/ToF-MS data included in the supplementary materials for this
%       manuscript. The code should be in the same directory as the data
%       files (i.e., the .csv files), and the user should run the code
%       using that location at the working directory. The code processes
%       the RP/ToF-MS data and produces several plots, including Figure 8
%       from the manuscript, and calculates the jet breakup parameters from
%       a fit of those data.
%
%       Note that the user must have the Matlab Curve Fitting toolbox
%       installed in order for this code to run


close all; clear; clc;
CONST.AMU_PER_KG = 6.022E26; CONST.C_per_q = 1.6022E-19;
```

## File I/O and User-Adjustable Settings

```
myMarkerStyle = 'ko';

% Path to directory containing data files
setup.DATA_PATH = '.\';

% List of data files to import
setup.DATA_FILES = {'1200.csv' '1250.csv' '1300.csv' '1350.csv' '1400.csv' ...
    '1450.csv' '1500.csv' '1550.csv' '1600.csv' '1650.csv' '1700.csv' ...
    '1750.csv' '1800.csv'};

setup.V_Emitter = 1500; % Emitter potential, Volts
setup.V_Extractor = 0; % Extractor potential, volts
setup.I_emitter_nA = 285; % Emitter current, nanoamps

setup.t_OFFSET = 2.1952e-04;
setup.BOUNDS = [1251 2500];

setup.TIA_GAIN = -4.8E6; % Transimpedance amplifier gain in Volts/Amp (negative because the TIA inverts the sign of the current)
setup.L_tof = 116E-3; % flight distance in meters

%{
Set bounds that determine what data get fit by error functions. The code
% will fit two error functions to the RP/ToF-MS data; one at 'low' m/q and
% another at 'high' m/q. The 'high m/q' region corresponds roughly to
% droplets, while the 'low m/q region' corresponds roughly to ions
%}
setup.high_amu_by_q_limits = [2E3 1E6];
setup.low_amu_by_q_limits = [100 10E3];

ToF_Data = loadToFData(setup);
```

## Process Data

```
% Compute m/q from time vectors
ToF_Data = getMassToChargeRatio(ToF_Data,setup);
```

## Fit error functions to high amu/q range droplets

Fit 'high m/q' (droplet) data to error functions in the time domain

```
ToF_Data = getErfFit(ToF_Data,setup);

for i=1:length(ToF_Data)
    cf_vals = coeffvalues(ToF_Data(i).high_mq.erfFit.result_pA);
    calcResults.high_mq.erfStepHeights_pA(i) = cf_vals(1);
    calcResults.high_mq.erfCenter_amu_by_q(i) = ...
        t_to_amu_by_q(cf_vals(3)/1E6,ToF_Data(i).V_RPA,setup.L_tof);
        ci = confint(ToF_Data(i).high_mq.erfFit.result_pA);
        calcResults.high_mq.erfCenter_amu_by_q_confint(:,i) = ...
            t_to_amu_by_q(ci(:,3)./1E6,ToF_Data(i).V_RPA,setup.L_tof);
    calcResults.high_mq.rsquare(i) = ToF_Data(i).high_mq.erfFit.gof.rsquare;

    cf_vals = coeffvalues(ToF_Data(i).low_mq.erfFit.result_pA);
    calcResults.low_mq.erfStepHeights_pA(i) = cf_vals(1);
    calcResults.low_mq.erfCenter_amu_by_q(i) = ...
        t_to_amu_by_q(cf_vals(3)/1E6,ToF_Data(i).V_RPA,setup.L_tof);
        ci = confint(ToF_Data(i).low_mq.erfFit.result_pA);
        calcResults.low_mq.erfCenter_amu_by_q_confint(:,i) = ...
            t_to_amu_by_q(ci(:,3)./1E6,ToF_Data(i).V_RPA,setup.L_tof);
    calcResults.low_mq.rsquare(i) = ToF_Data(i).low_mq.erfFit.gof.rsquare;
end
clear cf_vals ci;
```

## Finish loading calcResults structure with results

```
calcResults.V_RP = extractfield(ToF_Data,'V_RPA');
calcResults.V_Deficit = setup.V_Emitter - calcResults.V_RP; % Volage deficit, i.e. V_Emitter - V_RP
calcResults.high_mq.erfCenter_kg_by_C = calcResults.high_mq.erfCenter_amu_by_q ./ CONST.AMU_PER_KG ./ CONST.C_per_q;
    calcResults.high_mq.erfCenter_kg_by_C_confint = calcResults.high_mq.erfCenter_amu_by_q_confint ./ CONST.AMU_PER_KG ./ CONST.C_per_q;
calcResults.low_mq.erfCenter_kg_by_C = calcResults.low_mq.erfCenter_amu_by_q ./ CONST.AMU_PER_KG ./ CONST.C_per_q;
    calcResults.low_mq.erfCenter_kg_by_C_confint = calcResults.low_mq.erfCenter_amu_by_q_confint ./ CONST.AMU_PER_KG ./ CONST.C_per_q;
```

## Plot all data on same time x-axis with error function fits for high m/q range

This plot shows the 'RP/ToF-MS signal' vs time for each retarding potential. The RP/ToF-MS signal is calculated by taking the difference (i.e., subtracting) two ToF-MS signals taken at different retarding potentials

The black dashed curves are the fits calculated for each curve (fitted with error functions).

```
figure(); hold on;

for i=1:length(ToF_Data)
    plot(1E6.*ToF_Data(i).t,1E12*ToF_Data(i).y_t,'DisplayName',num2str(ToF_Data(i).V_RPA))

    % plot error function fit
    fitX = ToF_Data(i).high_mq.erfFit.t_us_data;
    fitY = ToF_Data(i).high_mq.erfFit.result_pA(fitX);
    plot(fitX,fitY,...
        'k--','HandleVisibility','off');
end
legend; grid on;
xlabel('Time (µs)'); ylabel('ToF1 - ToF2 (pA)');
xlim([0 250]);
title({'Raw RP/ToF-MS Signals vs. Time for Various Retarding Potentials', ...
    '(Black Dashed Lines are Erf Curve Fits)'});
```

## Plot all data on same m/q x-axis (kg/C)

Plot of all the RP/ToF-MS signals in the data set with mass-to-charge ratio on the x-axis. Each signal was found by converting the time domain RP/ToF-MS signal into the m/q domain using the appropriate retarding potential and Equation 2 from the manuscript

```
figure(); hold on;

for i=1:length(ToF_Data)
    plot(ToF_Data(i).massToCharge,1E12*ToF_Data(i).y_mq,'DisplayName',num2str(ToF_Data(i).V_RPA))
end
legend; grid on;
xlabel('Mass-to-Charge Ratio (kg/C)'); ylabel('ToF1 - ToF2 (pA)');
xlim([0 0.01]);
title('Raw RP/ToF-MS Signals vs. Mass-to-Charge Ratio for Various Retarding Potentials');
```

## Plot m/q center (center of erf fit) vs Retarding Potential

Plot the center m/q value determined from error function fits of the RP/ToF-MS signals (termed the 'average m/q') vs the retarding potential at which the RP/ToF-MS signal was measured

```
figure(); hold on;
scatter(calcResults.high_mq.erfCenter_kg_by_C,calcResults.V_RP,'o');
ylabel('Retarding Potential (V)'); xlabel('Center Mass-to-Charge Ratio (kg/C)');

% Add emitter potential to plot
myXlim = xlim();
plot([myXlim(1) myXlim(2)],setup.V_Emitter.*[1 1],'r--');

legend({'$\phi_\mathrm{RP}$ vs. $\bar{\zeta}$','Emitter Potential'},...
    'Interpreter','Latex','location','southeast');

title(['Retarding Potential $\phi_\mathrm{RP}$ vs average mass-to-charge ', ...
    '$\bar{\zeta}$'],...
    'Interpreter','Latex');
```

## Plot: Compare to GC '21 FIG 8 and Miller '21 Excess Potential

Compare our RP/ToF-MS data to the literature using the following method: Calculate the 'Excess Potential' (negative of the potential deficit) and plot results from our work and the literature. All data should follow a similar trend, despite the different emitter potentials used in the works. Our average mass-to-charge value is calculated from error function fits of our data. Only data points with an R-squared value greater than 0.4 are plotted for our data.

```
hf = figure('Units','inches');
hold on;
% % Uncomment to format figure
% hf.Color = [1,1,1];
% hf.Units = 'inches';
% tmpPosition = hf.Position;
% hf.Position = [0 0 3.25 3];

% ****************************************************************************
% Plot high m/q data from this work (with rsquare greater than 0.4)
% ****************************************************************************
ind = find(calcResults.high_mq.rsquare >= 0.4);

s = scatter(calcResults.high_mq.erfCenter_kg_by_C(ind),-calcResults.V_Deficit(ind),myMarkerStyle,'filled',...
    'DisplayName','This Work');
% Configure data tips
s.DataTipTemplate.DataTipRows(1).Label = "(X Data) Mass-to-Charge:";
s.DataTipTemplate.DataTipRows(2).Label = "(Y Data) Potential Excess:";
s.DataTipTemplate.DataTipRows(3) = dataTipTextRow("Retarding Potential:",calcResults.V_RP(ind));

% ****************************************************************************
% Add low m/q results from my data (with rsquare greater than 0.4)
% ****************************************************************************
ind = find(calcResults.low_mq.rsquare >= 0.4);
scatter(calcResults.low_mq.erfCenter_kg_by_C(ind),-calcResults.V_Deficit(ind),...
    myMarkerStyle,'filled','MarkerEdgeColor','k','HandleVisibility','Off');

% ****************************************************************************
% Add literature data
% ****************************************************************************
% Load literature data
[GM2021, Miller2021] = getLitData();

scatter(GM2021.fig8_300nA.kg_per_C,-GM2021.fig8_300nA.V_Deficit,'ksquare',...
    'DisplayName','GC et al. 2021')
scatter(GM2021.fig8_300nA.kg_per_C_low_mq,-GM2021.fig8_300nA.V_Deficit_low_mq,...
    'rsquare','MarkerEdgeColor','k','HandleVisibility','Off')

MillerStruct = Miller2021.EMI_IM_25eV_per_q_280pLps;
scatter(MillerStruct.kg_per_C,-MillerStruct.V_Deficit,...
    'k^','DisplayName','Miller et al. 2021');

% ****************************************************************************
% Finish formatting
% ****************************************************************************
ylabel('\phi_R_P - \phi_E'); xlabel('Mass-to-Charge (kg/C)');
grid on; legend('location','southeast');

xticks(0:0.001:0.006);
yticks(-400:100:400); ylim([-400 400]);
legend('fontsize',8);
set(gca,'fontsize',8);
box on;

title('Excess Potential vs. Average Mass-to-Charge Ratio (for R^2 >= 0.4)');

clearvars GM2021 Miller2021 ci tmp_ones s i j;
```

## Plot: Calculate jet velocity and breakup potential from my data

Plot retarding potential vs average mass-to-charge ratio as determined from error function fits of our RP/ToF-MS data.

The 'high m/q' data (i.e., the ToF signal associated with droplets in the plume) is fit by error functions. A linear fit is found for all data points where the r-squared of the fit is >= 0.4 and the average m/q is >= 1 g/C. That linear fit is used to calculate the jet breakup potential and breakup velocity according to Equation 8 in the manuscript. i.e., the fit slope is used to calculate breakup velocity and the y-offset is used to calculate the breakup potential

Also note Eqn 6 in Gamero-Castano 2021 (and the associated discussion): phi\_RP(i) = (1/2)\*v\_j^2\*m\_q(i) + phi\_j

```
hf = figure(); hold on;

% Find 'high m/q' data with a fit r-squared of at least 0.4 and m/q >= 1 g/C
ind = find(calcResults.high_mq.rsquare >= 0.4 & calcResults.high_mq.erfCenter_kg_by_C >= 0.001);
% Find 'high m/q' data with a fit r-squared >= 0.4 and m/q < 1 g/C
ind2 = find(calcResults.high_mq.rsquare >= 0.4 & calcResults.high_mq.erfCenter_kg_by_C < 0.001);

scatter(calcResults.high_mq.erfCenter_kg_by_C(ind),calcResults.V_RP(ind),...
    'ko','Filled','DisplayName','High m/q Data (m/q > 1 g/C)');
scatter(calcResults.high_mq.erfCenter_kg_by_C(ind2),calcResults.V_RP(ind2),...
    'ko','DisplayName','High m/q Data (m/q < 1 g/C)');

ylabel('Retarding Potential (V)'); xlabel('Center Mass-to-Charge Ratio (kg/C)');
grid on;

% Find linear fit
ft = fittype('poly1');
calcResults.jetBreakupFit = fit(calcResults.high_mq.erfCenter_kg_by_C(ind)',...
    calcResults.V_RP(ind)',ft);

% Plot linear fit alongside data
tmpX = calcResults.high_mq.erfCenter_kg_by_C(ind);
plot(tmpX,calcResults.jetBreakupFit(tmpX),[myMarkerStyle(1) '--'],'DisplayName','Linear Fit');
legend('location','northwest');

% Add my low m/q data to plot (w/ r^2 >= 0.4) (not included in fit)
ind = find(calcResults.low_mq.rsquare >= 0.4);
scatter(calcResults.low_mq.erfCenter_kg_by_C(ind),calcResults.V_RP(ind),...
    'k^',"filled",'DisplayName','Low m/q Data');

title({'Calculation of Jet Breakup Parameters from RP/ToF-MS Data',...
    '(For Data with R-Squared >= 0.4)'});

clearvars ind ind2 ft tmpX;
```

## Console output

Print text output to the console

```
fprintf('*****************************************************\n');
fprintf('Jet Breakup Fit:\n');
    tmp = coeffvalues(calcResults.jetBreakupFit);
fprintf('   Jet Breakup Velocity = %4.1f m/s\n',sqrt(2*tmp(1)));
fprintf('   Jet Breakup Potential = %4.1f V\n',tmp(2));
fprintf('   Jet Breakup Potential Deficit = %4.1f V\n',setup.V_Emitter - tmp(2));
    clearvars tmp;
```

```
*****************************************************
Jet Breakup Fit:
   Jet Breakup Velocity = 491.5 m/s
   Jet Breakup Potential = 1302.7 V
   Jet Breakup Potential Deficit = 197.3 V
```

## Functions

```
function [ToF_Data] = loadToFData(setup)

% Headers: (Row 3)
% time(s)  tof1(V)  tof2(V)  tof1-tof2(V)  trigger1(V) trigger2(V)
%
% Data begins on Row 4
for i=1:length(setup.DATA_FILES)

    ToF_Data(i).DATA_PATH = fullfile(setup.DATA_PATH, setup.DATA_FILES{i});
    ToF_Data(i).name = setup.DATA_FILES{i};
    ToF_Data(i).V_RPA = str2num(ToF_Data(i).name(1:find(ToF_Data(i).name=='.')-1));


    % *******************************************************
    %                   Load y vs. t data
    % *******************************************************
    % Read file
    data_opts = detectImportOptions(ToF_Data(i).DATA_PATH);
    data_opts.SelectedVariableNames = 1:6;
    DATA_MAT = readmatrix(ToF_Data(i).DATA_PATH,data_opts);

    % Restrict DATA_MAT to input file specified range
    DATA_MAT = DATA_MAT(setup.BOUNDS(1):setup.BOUNDS(2),:);

    % Load DATA_MAT data into ToF_Data structure
    ToF_Data(i).raw.t = DATA_MAT(:,1);
    ToF_Data(i).raw.tof1 = DATA_MAT(:,2);
    ToF_Data(i).raw.tof2 = DATA_MAT(:,3);
    ToF_Data(i).raw.tof1_tof2 = DATA_MAT(:,4);
    ToF_Data(i).raw.trigger1 = DATA_MAT(:,5);
    ToF_Data(i).raw.trigger2 = DATA_MAT(:,6);

    % Load processed data fields into ToF_Data structure
    ToF_Data(i).t = ToF_Data(i).raw.t - setup.t_OFFSET;
    ToF_Data(i).y_t = ToF_Data(i).raw.tof1_tof2 ./ setup.TIA_GAIN;

end


end

function [ToF_Data] = getMassToChargeRatio(ToF_Data,setup)
% Calculates mass to charge ratio from Equation 1 in Gamero-Castano's 2021
% ToF paper: "Electrosprays of highly conducting liquids: A study of droplet and ion
%       emission based on retarding potential and time-of-flight spectrometry
AMU_PER_KG = 6.022E26; C_per_q = 1.6022E-19;

for i=1:length(ToF_Data)
    t = ToF_Data(i).t;
    V = ToF_Data(i).V_RPA;
    L = setup.L_tof;

    ind = find(t>=0);
    ToF_Data(i).t_mq = t(ind);
    ToF_Data(i).y_mq = ToF_Data(i).y_t(ind);

    ToF_Data(i).massToCharge = 2*V*(ToF_Data(i).t_mq./L).^2;
    ToF_Data(i).massToCharge_amu_by_q = ...
        ToF_Data(i).massToCharge .* AMU_PER_KG .* C_per_q;
end

end

function [ToF_Data] = getErfFit(ToF_Data, setup)
% Function used to fit data with error function fits
%
% Note: Requires Matlab Curve Fitting Toolbox


% Notes: a*erf(b*(x-c))+d
%   Step rise in current due to large droplets occurs over a period of up
%   to half of the full m/q range at first glance
%       Erf(x) 10% to 90% rise occurs over a range of approx. dx=2.3
%           b min: 2.3E3 (for maximum step dx=1E-3 for 10% to 90% rise)
%           b max: 2.3E6 (for minimum step dx=1E-6 for 10% to 90% rise)
%       c expresses how far right the erf is centered
%
CONST.AMU_PER_KG = 6.022E26; CONST.C_per_q = 1.6022E-19;


% Erf Fit for High m/q range
for i=1:length(ToF_Data)

    t = ToF_Data(i).t;
    t(t<0) = 0;

    amu_by_q = (2 * ToF_Data(i).V_RPA .* t.^2/setup.L_tof.^2) ...
        .* CONST.AMU_PER_KG .* CONST.C_per_q ;

    % Restrict Erf fit to high m/q range
    ind = find(amu_by_q > setup.high_amu_by_q_limits(1) ...
        & amu_by_q < setup.high_amu_by_q_limits(2));

    t_us = t(ind).*1E6;
        ToF_Data(i).high_mq.erfFit.t_us_data = t_us;
        ToF_Data(i).high_mq.erfFit.amu_q_data = amu_by_q(ind);

    y = ToF_Data(i).y_t(ind) .* 1E12;
        ToF_Data(i).high_mq.erfFit.y_data = y;


    ft = fittype('a*erf(b*(t-c))+d', 'independent', 't', 'dependent', 'y' );
    options = fitoptions(ft);

    al = 0; au = 1E3; as = 1;
    bl = -inf; bu = inf; bs = 0.05;
    cl = t_us(1); cu = t_us(end); cs = 100;
    dl = -1000; du = 1000; ds = 0;

    %                          [  a     b       c    d]
    options.StartPoint =       [as bs cs ds];
    options.Lower =            [al bl cl dl];
    options.Upper =            [au bu cu du];

    [yfit,gof] = fit(t_us,y,ft,options);
    ToF_Data(i).high_mq.erfFit.result_pA = yfit;
    ToF_Data(i).high_mq.erfFit.gof = gof; %goodness of fit statistics
end

%Erf Fit for Low m/q range
for i=1:length(ToF_Data)

    t = ToF_Data(i).t;
    t(t<0) = 0;

    amu_by_q = (2 * ToF_Data(i).V_RPA .* t.^2/setup.L_tof.^2) ...
        .* CONST.AMU_PER_KG .* CONST.C_per_q ;

    % Restrict Erf fit to high m/q range
    ind = find(amu_by_q > setup.low_amu_by_q_limits(1) ...
        & amu_by_q < setup.low_amu_by_q_limits(2));

    t_us = t(ind).*1E6;
    ToF_Data(i).low_mq.erfFit.t_us_data = t_us;
    ToF_Data(i).low_mq.erfFit.amu_q_data = amu_by_q(ind);

    y = ToF_Data(i).y_t(ind) .* 1E12;
    ToF_Data(i).low_mq.erfFit.y_data = y;


    ft = fittype('a*erf(b*(t-c))+d', 'independent', 't', 'dependent', 'y' );
    options = fitoptions(ft);

    al = 0; au = 1E3; as = 100;
    bl = -inf; bu = inf; bs = 0.05;
    cl = t_us(1); cu = t_us(end); cs = 100;
    dl = -1000; du = 1000; ds = 0;

    %                          [  a     b       c    d]
    options.StartPoint =       [as bs cs ds];
    options.Lower =            [al bl cl dl];
    options.Upper =            [au bu cu du];


    [yfit,gof] = fit(t_us,y,ft,options);
    ToF_Data(i).low_mq.erfFit.result_pA = yfit;
    ToF_Data(i).low_mq.erfFit.gof = gof; %goodness of fit statistics

end

end

function [amu_by_q] =  t_to_amu_by_q(t_seconds,V_RPA,L_tof)
AMU_PER_KG = 6.022E26; C_per_q = 1.6022E-19;

kg_per_C = 2*V_RPA*t_seconds.^2/L_tof.^2;
amu_by_q = kg_per_C .* AMU_PER_KG .* C_per_q;

end

function [GM2021, Miller2021] = getLitData()
% Function to load literature data into structures (GM2021 and Miller2021
% structures) to more easily plot them

% *******************************************************************************************
% Gamero-Castano, 2021, "Electrosprays of highly conducting liquids: A study
%   of droplet and ion emission based on retarding potential and
%   time-of-flight spectrometry", Physical Review Fluids
% *******************************************************************************************
GM2021.fig8_300nA.V_Deficit = [102.78 48.24 -55.21 -155.38 -261.27 -370.39];
GM2021.fig8_300nA.kg_per_C = [0.001131514 0.001478908 0.00244665 0.003265509 0.004263027 0.005136476];
GM2021.fig8_300nA.V_Deficit_low_mq = [273.74];
GM2021.fig8_300nA.kg_per_C_low_mq = [0];

GM2021.fig8_400nA.V_Deficit = [390.8140168	245.0868486	180.7785108	101.7896266	31.74685792	-43.19615082	-145.8389316	-225.6236761];
GM2021.fig8_400nA.kg_per_C = [4.96278E-06	0.00098263	0.001379653	0.002004963	0.002615385	0.003334988	0.004337469	0.004878412];

GM2021.fig8_450nA.V_Deficit = [452.6026347	340.2138433	270.9951784	206.6232928	121.0688132	41.21547742	-75.30492848	-223.5982166];
GM2021.fig8_450nA.kg_per_C = [4.96278E-06	0.000957816	0.001513648	0.002223325	0.003151365	0.004029777	0.005310174	0.006913151];


% *******************************************************************************************
% Miller et al, 2021, "Capillary ionic liquid electrospray: beam
%   compositional analysis by orthogonal time-of-flight mass spectrometry",
%   Journal of Fluids Mechanics
% *******************************************************************************************

% From supplementary material, plot S4
Miller2021.EMI_IM_25eV_per_q_140pLps.label = 'Miller 2021, 0.14 nL/s';
Miller2021.EMI_IM_25eV_per_q_140pLps.V_Deficit = [224.2349077	199.1817629	174.1037684	148.9828514	123.8438618	99.42212855	73.36934299	48.12643592	22.92193314];
Miller2021.EMI_IM_25eV_per_q_140pLps.kg_per_C = [0.00049092	0.000504587	0.000548025	0.000642883	0.000759393	0.001016629	0.001227866	0.001468869	0.001663863];

% From supplementary material, plot S4
Miller2021.EMI_IM_25eV_per_q_280pLps.label = 'Miller 2021, 0.28 nL/s';
Miller2021.EMI_IM_25eV_per_q_280pLps.V_Deficit = [48.18	73.84	98.68	122.68	148.34	173.18	198.84	224.50	250.99	275.83	301.49];
Miller2021.EMI_IM_25eV_per_q_280pLps.kg_per_C = 1E-3.*[2.235	2.034	1.837	1.571	1.434	1.111	0.888	0.632	0.522	0.465	0.395];

end
```

Published with MATLAB® R2021a
