# Supplementary figures and images for: A simple retarding-potential time-of-flight mass spectrometer for electrospray propulsion diagnostics
Source: J Elect Propuls. 2023 Mar 31;2(1):13. doi: 10.1007/s44205-023-00045-y (PMC10066156; doi:10.1007/s44205-023-00045-y)

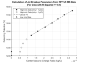

Supplement: Supplementary file 1 — Additional file 1. [file 44205_2023_45_MOESM1_ESM.zip › Data and MATLAB Code/RP ToF-MS Data/RP ToF Matlab Code (html version)/RP_TOF_Data_Analysis.png]

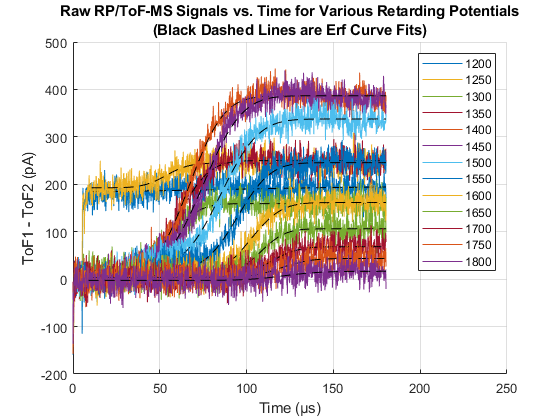

Supplement: Supplementary file 1 — Additional file 1. [file 44205_2023_45_MOESM1_ESM.zip › Data and MATLAB Code/RP ToF-MS Data/RP ToF Matlab Code (html version)/RP_TOF_Data_Analysis_01.png]

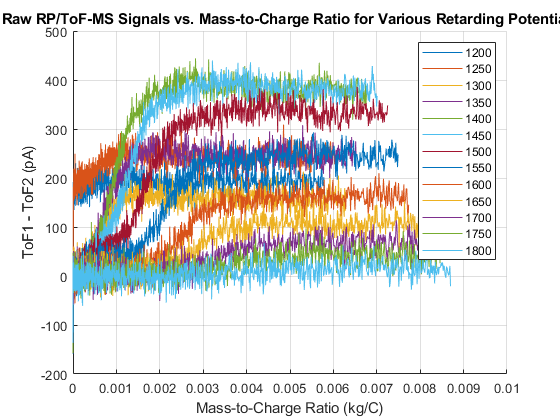

Supplement: Supplementary file 1 — Additional file 1. [file 44205_2023_45_MOESM1_ESM.zip › Data and MATLAB Code/RP ToF-MS Data/RP ToF Matlab Code (html version)/RP_TOF_Data_Analysis_02.png]

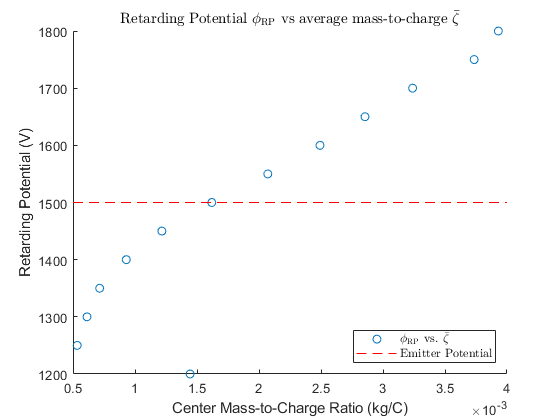

Supplement: Supplementary file 1 — Additional file 1. [file 44205_2023_45_MOESM1_ESM.zip › Data and MATLAB Code/RP ToF-MS Data/RP ToF Matlab Code (html version)/RP_TOF_Data_Analysis_03.png]

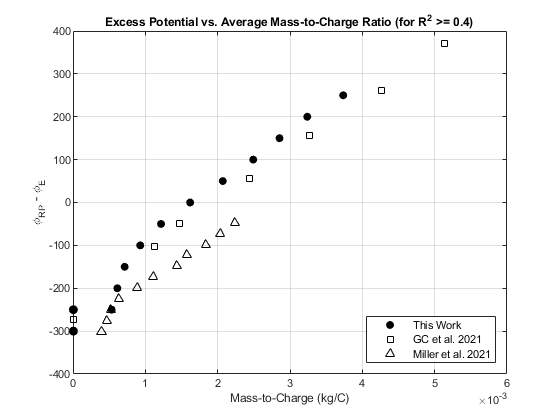

Supplement: Supplementary file 1 — Additional file 1. [file 44205_2023_45_MOESM1_ESM.zip › Data and MATLAB Code/RP ToF-MS Data/RP ToF Matlab Code (html version)/RP_TOF_Data_Analysis_04.png]

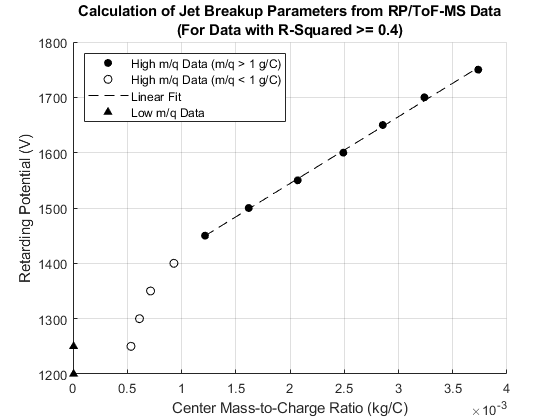

Supplement: Supplementary file 1 — Additional file 1. [file 44205_2023_45_MOESM1_ESM.zip › Data and MATLAB Code/RP ToF-MS Data/RP ToF Matlab Code (html version)/RP_TOF_Data_Analysis_05.png]
